# Translating international guidelines for use in routine maternal and neonatal healthcare quality measurement

**DOI:** 10.1080/16549716.2020.1783956

**Published:** 2020-07-13

**Authors:** Karen T. Chang, Puspita Hossain, Malabika Sarker, Dominic Montagu, Nirali M. Chakraborty, Andrea Sprockett

**Affiliations:** a Metrics for Management, Oakland, CA, USA; b James P. Grant School of Public Health, BRAC University, Dhaka, Bangladesh; c Heidelberg Institute of Global Health, University of Heidelberg, Germany

**Keywords:** Quality of care, measurement, maternal health, neonatal health, management, low- and middle-income, Bangladesh

## Abstract

**Background:**

Improving facility-based quality for maternal and neonatal care is the key to reducing morbidity and mortality rates in low- and middle-income countries. Recent guidance from WHO and others has produced a large number of indicators to choose from to track quality.

**Objective:**

To explore how to translate complex global maternal and neonatal health standards into actionable application at the facility level.

**Methods:**

We applied a two-step process as an example of how the 352 indicators in WHO’s 2016 Standards for Improving Quality of Maternal and Newborn Care in Health Facilities might be reduced to only those with the strongest evidence base, associated with outcomes, and actionable by facility managers. We applied Hill criteria and assessed whether indicators were within the control of facility managers. We next conducted a rapid review of supporting literature and applied GRADE analysis, retaining those with scores of ‘moderate’ or ‘high’. To understand the utility and barriers to measuring this limited set of indicators in practice, we undertook a case study of hypothetical measurement application in two districts in Bangladesh, interviewing 25 clinicians, managers, and other stakeholders.

**Results:**

From the initial 352 indicators, 56 were retained. The 56 indicators were used as a base for interviews. Respondents emphasized the practical challenges to the use of complex guides and the need for parsimonious and actionable sets of quality indicators.

**Conclusions:**

This work offers one way to move towards a reduced quality indicator set, beginning from current WHO guidance. Despite study limitations, this work provides evidence of the need for reduced and evidence-based sets of quality indicators if guides are to be used to improve quality in practice. We hope that future research will build on and refine our efforts. Measuring quality effectively so that evidence guides and improves practice is the first step to assuring safe maternal and neonatal care.

## Background

Through years of effort, and galvanized by the Millennium Development Goals, childbirth is becoming safer for both mother and child. The maternal mortality ratio (MMR) reduced globally by 44% from 2000 to 2015 [1], and the child mortality rate declined by more than 50% from 1990 to 2016 [2]. Much of this improvement has been due to increases in access to health services, improving rates of facility-based deliveries which are beneficial for both mother and newborn [3]. However, recent years have seen slowing progress, and the burden of maternal and neonatal deaths remains high in low- and middle-income countries (LMICs) [4]. To achieve the Sustainable Development Goals (SDGs) health targets, it is essential to focus on high-quality maternity health care [5]. Overall, the quality of maternal and neonatal healthcare provision is poor in LMICs, and there are significant variations across facilities [6]. Research has demonstrated that poor quality maternal health care contributes to higher levels of both maternal morbidities and mortalities, which have an important impact on a newborn’s ability to survive and to thrive [4]. Therefore, it is critical to improve the quality of services to reach these global targets and to improve the health and survival of both pregnant women and newborns.

Quality maternal and neonatal healthcare can encompass a range of services, including pregnancy and antenatal care, childbirth, post-partum care, and newborn care. Despite the quality of care domains defined by WHO and the Institute of Medicine [7,8], there is limited agreement on the definition of overall quality in healthcare received during labor, birth, and the postnatal period [9]. Currently, there is no agreement on what indicators should be nor what framework should be used for maternal and neonatal health. Among four global maternal and neonatal health-related initiatives, only 6 of 140 indicators overlap fully [10]. Adapting a standard global framework and identifying a set of indicators that are feasible to measure and, if improved, will result in better outcomes can help to guide stakeholders to improve the quality of maternal and neonatal health services. Policymakers, program leaders, and service providers need to better understand and measure quality [11,12], and what affects quality variations, in order to improve service delivery effectiveness and efficiency [12]. To do so, data is required from multiple sources from services at multiple levels, to inform the broader health information and policymaking sphere [13,14]. Data are collected at the lowest level of facilities, then aggregated up to district and eventually national level use [15], making health facility data a key part of policymaking. That information can be used to inform providers at a community, health facility, district, and national level [16].

Recent initiatives related to the improvement of maternal and neonatal healthcare include the WHO’s *Standards for Improving Quality of Maternal and Newborn Care in Health Facilities*, a complement to the WHO and UNICEF *Every Mother Every Newborn (EMEN) Standards for Quality Improvement* [17].

The *Standards for Improving Quality of Maternal and Newborn Care in Health Facilities* presents the most comprehensive set of indicators with a broad audience to measure progress towards achieving good quality of care. The indicators were created for use by a broad audience including policymakers, program managers, national, subnational, district, and facility-level health planners, healthcare providers, professional bodies, and technical partners collaborating with LMIC ministries of health, and in training medical professionals, and include 352 input, output, and outcome indicators. Input and output indicators, or measures of the resources required to provide care and the extent to which the process of care provision was as expected [18], provide an important source of routine feedback, allowing program managers to adjust service provision in order to ultimately achieve long-term health outcomes. Outcome measures demonstrate the effect of an intervention and included people centered and health outcomes [18].

Measuring a large set of indicators at lower-level facilities in LMICs may be challenging and resource prohibitive [19]. The current Quality of Care Network is using a multi-stakeholder and well-resourced approach to ensuring that these standards can be implemented, and measured, at the national level, and that quality can be measurably improved at the facility level across participating facilities in the nine project countries, including Bangladesh where our research was conducted [20]. Bangladesh has long prioritized investments to improve the quality of care at all levels of its health system, including maternal and neonatal healthcare [20,21]. Yet the lack of appropriate metrics for QoC measurement remains a challenge [22]. While many of the WHO QoC indicators may be suitable and desirable to define and support good practices, some indicators may not be useful in providing actionable guidance on minimum standards from the perspective of routine monitoring and adaptive management at the facility level. Better data and better methods to routinely measure quality are needed in order to develop and implement effective solutions. Indicators for all aspects of maternal and newborn healthcare should be evidence-based, associated with important maternal and newborn health outcomes that can be influenced by provider actions, easy to measure reliably and across various settings, effective at differentiating between good and poor care, acceptable to healthcare providers, and affordable to implement [21]. Yet large lists of maternal and neonatal (MNH) related indicators exist, and recent research has shown that defining a feasible set of evidence-based indicators continues to be a challenge [23,24].

Using a proof of concept approach, this study explores one method to translate complex guidelines and standards into actionable application at lower levels of the health system. A successful application of the method has the possibility to be scaled up, resulting in sufficient data to suggest a parsimonious and feasible set of MNH quality indicators for routine collection in low resource settings.

## Methods

We sought to demonstrate how to reduce a large set of indicators to those most likely to be causally related to improved health outcomes and supported by the best quality of evidence. Using a reduced indicator list, we then explored the perspectives of in-country stakeholders to understand which might be relevant to them, and thus to improve provider and manager buy-in. To guide our process to reduce and prioritize the indicators found in the WHO QoC standards, we utilized a three-step process: 1) an assessment of the link between an indicator and intended outcomes through the application of Bradford Hill’s causality criteria and whether indicators were within the control of a facility manager; 2) a rapid review of supporting literature and an evaluation of the quality of evidence using a GRADE analysis; and 3) an exercise in Bangladesh to ask stakeholders to describe the utility and feasibility of incorporating this reduced set of quality indicators into routine measurement, presented as a case study of hypothetical measurement application.

In understanding the role of quality indicators to ensure positive health outcomes, we use Donabedian’s framework, which posits that there are structural, process, and outcome aspects of quality. Structural features encompass the context in which care is provided, while process features include provision of care. Structure and process aspects both influence health outcomes [25]. Measures of outcome quality cannot guide improvement but do indicate that a failure may have occurred along the health-production pathway. In the first step of our indicator prioritization, we excluded all 78 outcome indicators from the 352 indicators published by the WHO since these are an ex-post-facto assessment of whether quality was provided.

### Assessment of causality and actionability

Next, we assessed whether each of the remaining 274 input and output indicators measures something that could be causally linked to an improved maternal or newborn health outcome. In other words, an improvement in the indicator can plausibly lead to an improvement in an outcome. For this, we applied Bradford Hill’s causality criteria [26]. Hill offered nine criteria as follows: strength of the association, consistency, specificity, temporality, dose-response, plausible mechanism, coherence, experimental evidence, and analogy. In our assessment, the criterion of plausibility was given greater weight, assessing if there is a known biological explanation or plausible explanation for how the exposure of interest (quality indicator) might result in or contribute to an outcome of interest.

Indicators deemed outside of the control of the facility manager were also excluded at this stage as they could not be addressed at the facility level, and so were analogous to outcome indicators for the purpose of this study: indicative of failure, but not guiding improvement.

Two senior investigators (DM and MS) independently conducted the subjective assessment to determine if an association between indicator and outcome is due to causation. Differences were adjudicated through verbal discussion between the two investigators. Items for which there was consensus that an indicator did not meet multiple criteria were excluded, and where there was disagreement or uncertainty, the indicator was retained.

### Rapid review and GRADE analysis

**Figure 1.**
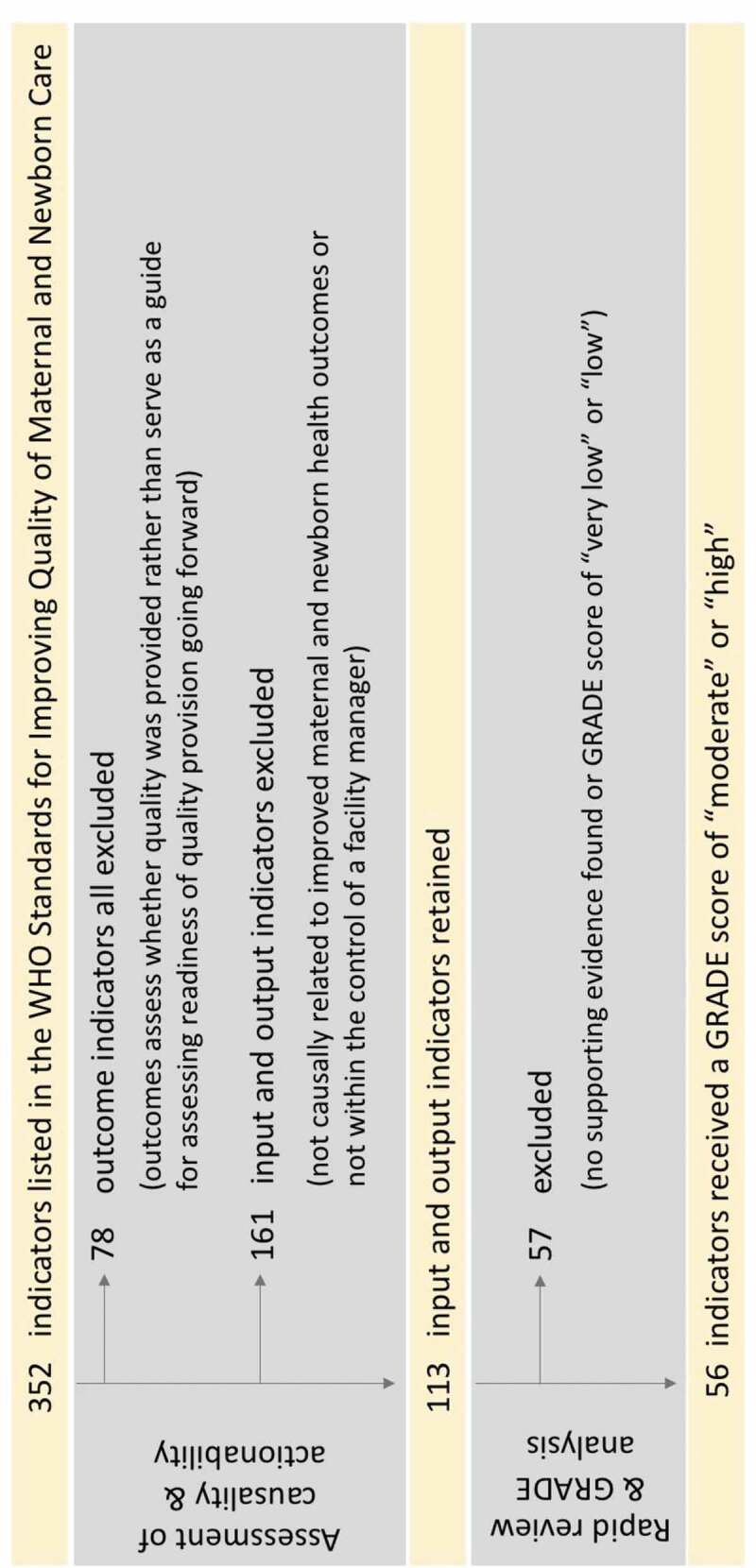
Flowchart of indicator exclusion process.

Following the application of the causality criteria, a second stage of reduction was undertaken. Three investigators (KC, PH, AS) conducted a rapid review of the literature supporting each indicator, with an emphasis on research conducted in LMIC settings. Rapid reviews offer a streamlined alternative to systematic reviews and allow for synthesizing evidence in a timely manner [27]. Where applicable, existing systematic reviews, like those identified from The Cochrane Library, were prioritized over searching for articles from individual studies [28]. Two investigators (KC and PH) subsequently performed a GRADE (Grading of Recommendations, Assessment, Development and Evaluations) analysis following an approach adapted from BMJ Best Practice [29]. Following GRADE methodology, systematic reviews and randomized controlled trials begin as high-quality evidence, while observational studies begin as low-quality evidence. Studies can then be downgraded based upon limitations in the study design and execution leading to the risk of bias, imprecision in the effect, inconsistency in the effects, or indirectness of evidence [30]. The evidence presented in these studies received one of four ratings – high, moderate, low, or very low, for the quality of the evidence linking the quality indicator to maternal and newborn health outcomes. Indicators with GRADE scores of ‘high’ or ‘moderate’ were retained. Those with GRADE scores of ‘low’ or ‘very low’, or those for which we could find no supporting evidence, were removed (Figure 1).

### Case study

Having created a reduced list of WHO indicators applicable for use in LMICs which may be within the control of a facility manager, and which have reasonable evidence, we sought to understand the feasibility of their routine implementation, as well as other challenges and considerations in quality measurement. The conversations with stakeholders focused on understanding current quality systems for maternal and newborn care at health facilities, and whether incorporating the shortened list of WHO standard indicators would be useful and feasible. We interviewed key stakeholders in Bangladesh using semi-structured qualitative interviews that focused on the reduced set of indicators. The interview guide (Supplementary File Interview Guide) contained three thematic sections, each of which included open-ended questions. Interviewers attempted to complete the guide in order, but were cognizant of interviewee time limitations, and cut short the first (General Information) section, where needed. The second section directed the interviewer to ask the interviewee if each indicator within our shortened list is measured, how and if it is useful, or why it is not collected. The third section allowed the interviewee to describe other data collected by the facility, and general comments on quality monitoring.

A range of physicians, nurses, program managers at international NGOs, donors, academics, and a representative of the Ministry of Health and Family Welfare (MoHFW) based in urban Dhaka and Khulna districts were purposively selected (Table 1). Khulna is located approximately 300 km southwest of Dhaka. Dhaka district was selected for the presence of donors, academic institutions, NGO headquarters, and tertiary health facilities, while Khulna was selected because it was reported to have the lowest maternal mortality ratio across all districts in Bangladesh [31]. Participants were conveniently selected and were approached with a copy of the consent form explaining the purpose of the study and given the opportunity to ask any questions. Only those who provided written informed consent were interviewed. Participants privately spoke to investigators (KC and PH) in English or Bengali, in the presence of a note-taker. Interviews were audio-recorded when participants allowed and transcribed verbatim, and then translated into English. If participants declined an audio-recording of the interview, the investigator made verbal notes summarizing the interview upon its conclusion and the recorded summary was used for analysis.

**Table 1.** Participant summary.

Participant type	No. of interviews	Male	Female
International non-governmental organization (INGO) representative	2	2	-
Researcher at an academic/research institution	3	2	1
Ministry of Health and Family Welfare (MoHFW) representative	1	1	-
Advisor at a donor organization	1	-	1
Physician at a tertiary level academic hospital	2	1	1
Senior physician at a tertiary level public hospital	2	1	1
Mid-level physician at a tertiary level public hospital	3	-	3
Physician at a tertiary level private hospital	2	1	1
Physician at a private practice	2	1	1
Physician at a secondary level public facility	3	3	-
Nurse at a secondary level public facility	3	-	3
Physician at a primary level public facility/Upazila Health Complex (UHC)	1	1	-
TOTAL	25	13	12

Data analysis was completed by the two investigators (KC and PH) who conducted the interviews and were most immersed in the raw data. Interviews were analyzed using thematic analysis to capture the detail and complexity of the interview responses [32,33]. Each of the two investigators reviewed all transcripts closely to familiarize themselves with the data. The analysis relied on both deductive and inductive approaches to capture all relevant information. Initial codes were informed deductively by the interview guide and the categories of indicators following the GRADE analysis. Codes were first generated based on aspects of the data that most related to the research objectives. Initial codes were applied to each transcript by one of the two investigators. After the initial coding, the investigators reviewed the interviews using an inductive process to create additional codes and identify nuances that emerged from the analysis. Codes were then reviewed and refined to minimize duplicate or redundant codes. An example quote, code, and theme are provided below:

**Table:** 

Quote	Code	Theme
It’s necessary but who will do that? Like ‘the proportion of all newborns in the health facility who received a full clinical examination before discharge’ – how will I keep this proportion? … if I tell the proportion, how will I get that? Who will get that? It is really tough. To measure this, you have to record the details. That is very tough …”	Routine indicators are not feasible	Challenges in monitoring quality

Each interview was re-read to edit the analysis using the refined set of codes. Memo writing was conducted to aid in summarizing and reflecting on concepts and patterns during the coding process. Network analysis was used to visually explore how codes were related and to identify overarching themes. Data analysis was conducted using ATLAS.ti version 8.

## Results

### Assessment of causality and actionability

Figure 1 presents a flowchart of the indicator exclusion process across each stage. All 78 outcome indicators (representing 22% of all indicators) were excluded in the first stage. For the 161 (46%) input and output indicators that were excluded, verbal agreement between two investigators had been achieved that an indicator would either not be causally related to improved maternal and newborn health outcomes or not be within the control of a facility manager. Multiple reasons for exclusion may have been cited for an individual indicator. Among the input and output indicators excluded, 32 (20%) lacked specificity, 55 (34%) lacked temporality, 3 (2%) did not show a biological gradient, 56 (35%) lacked plausibility, 83 (52%) lacked coherence, and 71 (44%) were beyond the control of a facility manager. A total of 113 (32%) of the total 352 indicators were retained. Supplementary Table 1 lists all 352 WHO QoC indicators and notes the reason for exclusion, if applicable.

### Rapid review and GRADE analysis

For the 113 retained indicators from the first phase, a rapid review of existing literature returned no supporting evidence for 22 (19%) indicators. The majority of the indicators for which no evidence was found were input indicators describing the presence of clinical protocols in the health facility. In these cases, there may be evidence that adherence to protocols resulted in improved health outcomes; however, none that suggested the presence of protocols in a facility alone affected outcomes. Adherence to protocols would be considered output or process indicators; no such indicators were included as part of the WHO QoC Standards.

From the GRADE analysis of supporting literature identified in the rapid review for the remaining input and output indicators, 12 (11%) indicators had a score of ‘very low’ and 19 (17%) indicators had a score of ‘low.’ Four of the indicators with a GRADE score of ‘moderate’ were duplicates and were dropped. At the end of our review process, 56 of 352 indicators were retained. Of the 56 indicators that were retained, 38 had a GRADE score of ‘moderate’, and 18 had a GRADE score of ‘high.’ Supplementary Table 2 lists each input and output indicator reviewed at this stage, supporting literature identified, and the criteria used to assess the quality of evidence.

### Case study

Table 1 summarizes the types of participants interviewed in Bangladesh. Of the 25 interviews, five were conducted in English, and 20 were conducted in Bengali. Of note, only 13 participants allowed interviews to be recorded; for these interviews, verbatim transcripts were analyzed. Ten of the 12 participants who did not allow interviews to be recorded were staff at government facilities; one was an MOHFW representative and one was an advisor at a donor organization. Interviews with staff at facilities, both public and private, were often no longer than 30 minutes as they had to return to their clinical duties.

Overall, stakeholders in Bangladesh recognized a shift in focus toward improving the quality of healthcare services is needed to continue making gains in reducing maternal and neonatal mortality. However, in terms of the feasibility and utility of implementing WHO QoC indicators routinely, they cited several factors to be considered within the current context of healthcare delivery in Bangladesh. Each quote includes the participant’s position, type of organization, and level of health facility, if applicable and where verbatim recordings are available.

#### Usefulness of QoC standards & indicators

Given the current systemic challenges in service delivery that are well recognized in LMICs, participants stated that having indicators to guide quality of care improvement is useful as it sets a standard and allows for monitoring of programs and performance. A researcher explained that having a standardized QoC tool is useful to hold people accountable and to provide motivation for staff to improve their performance. A QoC tool might then address concerns shared by a representative from the MOHFW who described a high rate of government sector worker absenteeism and the need for proper monitoring and supervision. These standards require a regular supervisory structure be maintained that would ultimately benefit staff morale and accountability in facilities.

Most of us, we do it, we have a job, we get paid for it … you know, there’re, there is certain level of motivation to produce good work. What drives the motivation? One of the things that drives the motivation is that you do want to see that your boss is happy. Right? It, especially in a bureaucracy, you know, and the public system, that’s a major driving force. So, if your boss never turns up and asks you, asks you, how is the work, never comes around and sees what you’re doing, why would you work? Why would you care to produce good outputs? You wouldn’t. So, I think for me, the biggest thing is that there’s absolute, zero-level accountability in many of these low-level facilities … things could be a lot better, even despite those challenges, if the accountability was in a better shape. (Researcher, academic/research institution)

In contrast, a physician at a secondary level public facility argued that the WHO indicators may lead to unfair judgements about the performance of a facility. He asserted that these indicators do not take into account the shortage in per capita investment and the lack of manpower and resources. He emphasized that any failings would not necessarily be the fault of the manager at a single facility if these larger systemic issues can only be addressed at higher levels of the government.

#### Resources needed for routine measurement of QoC indicators

The same barriers that currently hinder service delivery overlap with the challenges participants identified when asked if routine quality measurement could be feasibly built into the current health system in Bangladesh. Participants mentioned that recording quality data requires designated personnel, which most facilities currently lack. At many of the facilities, the registers are maintained by sisters-in-charge (nurses) or a physician, which is often an activity they perform in addition to their clinical workload. If additional data are required for routine indicators, designated quality improvement personnel are needed to collect this type of data and ensure that it is accurate. One physician at a tertiary level public facility explained, ‘If you say that you’ll visit some facility to measure how many babies were clamped within 1–3 minutes, you’ll get some document, but that is not the reality.*’*

A participant at a tertiary level academic hospital explained that current registers were generally focused on clinical data points and include information related to the date of admission, primary and final diagnosis, the treatment plan, and the cause of patient mortality. These records are kept in patient files and registers within the respective departments. The participant expressed the importance of maintaining such records as they contribute to national statistics through the District Health Information System 2 (DHIS2). When asked about current data systems, another physician at a tertiary level academic facility stated, ‘Before we didn’t have any information database. Now we are giving data input day-to-day through MIS [Management Information System] section of DG [Directorate General] health; it is being disseminated and everyday data is going to the DG office. The data are being input according to ICD [International Classification of Diseases] code. We have a delegated personnel for this; he is doing these daily.’

#### Indicators should be prioritized

All participants agreed that the list of 56 indicators was still too long to be measured routinely. Many of them stated that the indicators should be prioritized given the existing strain on resources to deliver care, much less to collect data to monitor quality (Supplementary Table 3). For example, as one participant explained, while he thought the output indicators related to emergency and immediate newborn care were necessary to record, he was concerned with who would maintain these records.

It’s necessary but who will do that? Like ‘the proportion of all newborns in the health facility who received a full clinical examination before discharge’ – how will I keep this proportion? … if I tell the proportion, how will I get that? Who will get that? It is really tough. To measure this, you have to record the details. That is very tough …. (Physician, tertiary level public hospital)

## Discussion

With quality of care now in the forefront [34], the creation of the *WHO Standards for Improving Quality of Maternal and Newborn Care in Health Facilities* to guide efforts in quality measurement is a welcome addition to global guidance. However, for the purpose of routine measurement for quality improvement at the facility level, reducing this set of indicators to an actionable, shortened list feasible for implementation is a required first step. Furthermore, indicators that will be collected and used by facility managers, and used to feed up into national policy, should incorporate the perspectives of those service providers.

Others have also recognized the need to more closely examine and refine the wide-array of proposed indicators in maternal and neonatal health. In a scoping review of maternal and newborn indicators, authors found that of the 140 indicators included, about 25% required clearer definitions and further development [10]. They concluded that the volume of existing indicators overwhelms national and local leaders interested in establishing monitoring systems and that more effort is required to better harmonize indicators across the numerous global monitoring initiatives [10,35]. A recent systematic review of indicators related to maternal and child healthcare reported that of the 1791 indicators identified, only 6.7% were evidence-based, reliable, and demonstrated to be feasible [36]. Despite this high volume of existing indicators, authors cited gaps in the continuum of care, including those covering the postpartum period, especially for the mother, those focused at the primary care level, and those with specific considerations for LMIC settings [36]. Work to understand the validity of MNH indicators demonstrated that indicators that are meaningful to users, and reflect the reality of service provision, will lead to quality of care improvements, as well as improvements in MNH health outcomes [23].

Following the initial reduction process, we then explored the perspectives of in-country stakeholders on our shortened list of 52 indicators. This is a first attempt to understand the usefulness and feasibility of the WHO indicators through interviews with stakeholders in Bangladesh. Participants in our study agreed that a set of metrics to guide quality improvement is necessary; however, the current WHO indicators create a burden of measurement that may only work to overwhelm the current health system in Bangladesh. In an ongoing study in Bangladesh, Ghana, and Tanzania, an assessment of facility readiness for implementing the WHO QoC Standards may help identify existing barriers to introducing quality improvement interventions in health systems in LMICs [37]. Citing systemic challenges in service delivery, participants commented on the need to prioritize indicators within the reduced list even further. Work with international MNH measurement experts further supports the need to prioritize fewer indicators that are locally relevant and will lead to action [23]. Additionally, differentiating between the facility and systemic issues that may impact performance as assessed by these indicators may help alleviate concerns that quality measurement may become a tool for blame.

This study has several limitations. A combination of applying Hill’s causal criteria and assessing whether indicator performance was within the control of a facility was used in the first stage of indicator reduction. This subjective assessment excluded indicators that are listed in other WHO recommended guidelines, such as the WHO Safe Childbirth Checklist [38]. Such checklists include important measures of the larger health systems required to ensure safe childbirth. However, these were determined, in our process, to be less well adapted as a guide for self-assessment by the health facility; a result with which other researchers may disagree. In addition, a rapid review rather than a systematic review of the literature was used to assess the quality of evidence in the interest of time and resources. A systematic review of the literature available for each of the 352 indicators would have required resources that were well beyond that allotted for this activity; however, we acknowledge that a systematic review approach would have generated a stronger body of evidence and we encourage others to carry out this activity. In addition, our interviews with stakeholders in Bangladesh were restricted to those located primarily in urban settings. We recognize that those in rural settings may have different priorities and barriers. Approximately half of the participants refused to allow us to record the interviews, and in these cases, we relied heavily on the interview notes and the interviewer’s recollection of content when recording herself immediately following the interview. Although we did not probe why participants declined to be recorded, we hypothesize that these participants may have had concerns about repercussions or confidentiality. Interviews were conducted for participant convenience at their place of work. It is possible that participants may have been more willing to record the interviews in a different setting. Finally, in reflecting on these findings, it is important to acknowledge that this formative research was conducted in only one country. The priorities and barriers to high quality of care identified in these interviews may be different in other countries; however, we anticipate that the need to prioritize indicators, especially those with a strong evidence-base that can be correlated with improved health outcomes, could be generalized to other settings that also experience a strain on existing health systems.

## Conclusion

The recent global recognition of the need for high quality of care to facilitate advancements in maternal and neonatal health outcomes has amplified efforts to provide guidance for LMIC service provision. Yet to effectively measure and improve quality, indicators need to reflect the needs of service providers striving to provide the best quality service for their patients. They must also take into account the realities of often limited resources for data collection, analysis, and application. This proof-of-concept for translating international guidelines for use in routine quality monitoring at lower levels of the health system in LMICs demonstrates how to reduce the number of indicators and to explore the real-world utility of indicators from this reduced set that are most likely linked to improved health outcomes. This outcomes-oriented approach for guiding reduction of indicators may allow for increased buy-in by providers, decrease the burden associated with measurement, and increase the likelihood of effective implementation into routine processes that could, therefore, improve the quality of care. Future work is needed to demonstrate that facility performance as measured by priority quality of care indicators is linked to improved health outcomes of mothers and their newborns.

## Supplementary Material

Supplemental Material

## Paper context

Improving facility-based quality for maternal and neonatal care is key to reducing morbidity and mortality rates in low- and middle-income countries. Recent guidance from WHO and others have produced a large number of indicators to choose from to track quality. This work offers one way to move towards a reduced quality indicator set. Additional work is required to develop a set of evidence-based indicators that are actionable at the facility level to improve key maternal health outcomes.

## Supplementary material

The supplemental data for this article can be accessed here.
