# Supplementary material for: Translating international guidelines for use in routine maternal and neonatal healthcare quality measurement
Source: Glob Health Action. 2020 Jul 13;13(1):1783956. doi: 10.1080/16549716.2020.1783956 (PMC7480423; doi:10.1080/16549716.2020.1783956)
Supplement: Supplemental Material [file ZGHA_A_1783956_SM0135.zip › MHQ paper_SupplementaryFileTable1_8May2020.docx]

## **Supplementary Data Table 1: Status at first stage for each of the 352 indicators from the 2016 WHO Standards for Improving Quality of Maternal and Newborn Care in Health Facilities report and reason for exclusion**

|  |  |  |  |  |  | **Reason indicator dropped in first stage** | | | | | | |
| --- | --- | --- | --- | --- | --- | --- | --- | --- | --- | --- | --- | --- |
| **QS#** | **Quality Statement** | **Ind#** | **Indicator** | **Indicator Type** | **Excluded in first stage** | **Outcome indicator** | **Lacked specificity** | **Lacked temporality** | **Lacked biological gradient** | **Lacked plausibility** | **Lacked coherence** | **Beyond control of facility manager** |
| 1.1a | Women are assessed routinely on admission and during labour and childbirth and are given timely, appropriate care. | 1 | The health facility has the basic essential equipment and supplies for routine care and detection of complications (thermometers, sphygmomanometers, fetal stethoscopes, urine dipsticks) available in sufficient quantities at all times in the areas of the maternity unit for labour and childbirth. | Input | No | No | No | No | No | No | No | No |
|  |  | 2 | The health facility has written, up-to-date clinical protocols for assessing intrapartum care and action in the labour and childbirth areas of the maternity unit that are consistent with WHO guidelines. | Input | No | No | No | No | No | No | No | No |
|  |  | 3 | Health-care staff in the labour and childbirth areas of the maternity unit receive in-service training and regular refresher sessions at least once every 12 months in the identification and management of obstetric emergencies during labour and childbirth. | Input | Yes | No | Yes | No | No | No | No | No |
|  |  | 4 | Health-care staff in labour and childbirth areas receive at least monthly drills or simulation exercises and supportive supervision in routine care and detection of obstetric complications during labour and childbirth. | Input | Yes | No | Yes | No | No | No | No | No |
|  |  | 1 | The proportion of all women who gave birth in the health facility whose blood pressure, pulse and temperature were appropriately recorded during labour, childbirth and the early postpartum period (and acted on if appropriate). | Output | Yes | No | Yes | No | No | No | No | No |
|  |  | 2 | The proportion of all women who gave birth in the health facility who received oxytocin within 1 min of the birth of their baby. | Output | No | No | No | No | No | No | No | No |
|  |  | 3 | The proportion of all women who gave birth in the health facility whose progress in labour was correctly monitored and documented with a partograph and a 4-h action line. | Output | Yes | No | Yes | No | No | No | No | No |
|  |  | 4 | The proportion of all women who gave birth in the health facility whose urinalysis result was appropriately recorded during labour, childbirth and the early postpartum period (and acted on if appropriate). | Output | Yes | No | Yes | No | No | No | Yes | No |
|  |  | 5 | The proportion of all women who gave birth in the health facility who received any option for pain relief during labour and childbirth. | Output | Yes | No | No | No | No | Yes | No | No |
|  |  | 1 | The health facility perinatal mortality rate [number of foetal death (stillbirths) or early neonatal deaths / the total number of births of babies weighing at least 1000 g or of 28 weeks’ gestation (stillbirths + live births) x 1000]. | Outcome | Yes | Yes | N/A | N/A | N/A | N/A | N/A | N/A |
|  |  | 2 | Intrapartum stillbirth rate (number of stillbirths occurring during the intrapartum period per 1000 births). | Outcome | Yes | Yes | N/A | N/A | N/A | N/A | N/A | N/A |
|  |  | 3 | The proportion of all women admitted to the health facility in active labour who gave birth within 12 h. | Outcome | Yes | Yes | N/A | N/A | N/A | N/A | N/A | N/A |
| 1.1b | Newborns receive routine care immediately after birth. | 1 | The health facility has written, up-to-date, clinical protocols for essential newborn care that are consistent with WHO guidelines and are available in the labour and childbirth areas of the maternity unit. | Input | No | No | No | No | No | No | No | No |
|  |  | 2 | The health facility has supplies of sterile cord ties (or clamps) and scissors (or blades), available in sufficient quantities at all times for the expected number of births. | Input | No | No | No | No | No | No | No | No |
|  |  | 3 | The health facility has supplies of clean towels in the labour and childbirth areas for immediate drying of newborns, available in sufficient quantities at all times for the expected number of births. | Input | Yes | No | Yes | No | Yes | No | No | No |
|  |  | 4 | Health-care staff in the labour and childbirth areas of the maternity unit receive in-service training or regular refresher sessions in essential newborn care and breastfeeding support at least once every 12 months. | Input | Yes | No | Yes | No | No | No | No | No |
|  |  | 5 | The health facility has local arrangements and a mechanism to maintain a documented room temperature in the labour and childbirth areas at or above 25 °C and free of draughts. | Input | Yes | No | Yes | No | No | No | No | No |
|  |  | 6 | Health-care staff in the labour and childbirth areas receive at least monthly drills or simulation exercises and supportive supervision in essential newborn care and supporting breastfeeding. | Input | Yes | No | Yes | No | No | No | No | No |
|  |  | 1 | The proportion of all newborns who were breastfed within 1 h of birth. | Output | No | No | No | No | No | No | No | No |
|  |  | 2 | The proportion of all newborns who were kept in skin-to-skin contact (with body and head covered) with their mothers for at least 1 h after birth. | Output | No | No | No | No | No | No | No | No |
|  |  | 3 | The proportion of all newborns who received all four elements of essential newborn care: immediate and thorough drying, immediate skin-to-skin contact, delayed cord clamping and initiation of breastfeeding in the first hour. | Output | Yes | No | No | No | No | No | No | Yes |
|  |  | 4 | The proportion of all newborns whose umbilical cord was clamped 1–3 min after birth. | Output | No | No | No | No | No | No | No | No |
|  |  | 5 | The proportion of all newborns who were dried immediately and thoroughly at birth. | Output | No | No | No | No | No | No | No | No |
|  |  | 1 | The proportion of all newborns who had a normal body temperature (36.5–37.5 °C) at the first complete examination (60–120 min after birth). | Outcome | Yes | Yes | N/A | N/A | N/A | N/A | N/A | N/A |
| 1.1c | Mothers and newborns receive routine postnatal care. | 1 | The health facility has written, up-to-date clinical protocols for postnatal care in the maternity and/ or postnatal care areas of the maternity unit that are consistent with WHO guidelines. | Input | No | No | No | No | No | No | No | No |
|  |  | 2 | The health facility practices and enables rooming-in to allow mothers and babies to remain together 24 h a day. | Input | No | No | No | No | No | No | No | No |
|  |  | 3 | The health facility has a written breastfeeding policy that is routinely communicated to all health care and support staff. | Input | Yes | No | Yes | No | No | Yes | No | No |
|  |  | 4 | Health-care staff in the maternity unit receive in-service training and regular refresher sessions in routine postnatal care and breastfeeding at least every 12 months. | Input | Yes | No | Yes | No | No | No | No | No |
|  |  | 5 | The health facility has local arrangements to ensure that every mother knows when and where postnatal care for herself and her newborn will be provided after discharge from the hospital. | Input | Yes | No | Yes | No | Yes | No | Yes | No |
|  |  | 6 | The health facility has local arrangements for alternative feeding methods, including cup or cup-and- spoon feeding, and avoids bottle-feeding. | Input | Yes | No | Yes | No | No | No | No | No |
|  |  | 7 | The health facility has local arrangement to inform pregnant women and their families about the benefits and management of breastfeeding. | Input | Yes | No | Yes | No | Yes | No | Yes | No |
|  |  | 8 | The health facility ensures that feeding of infant formula is demonstrated to mothers and family members of newborns only when needed, with a full explanation of the hazards of improper use. | Input | Yes | No | Yes | No | No | No | No | No |
|  |  | 1 | The proportion of all newborns on postnatal care wards or areas in the health facility who received vitamin K and full vaccination as per national guidelines. | Output | No | No | No | No | No | No | No | No |
|  |  | 2 | The proportion of all stable newborns in the health facility who are fed exclusively on breast milk from birth to discharge. | Output | Yes | No | No | Yes | No | No | No | Yes |
|  |  | 3 | The proportion of all women in postnatal care wards or areas in the health facility who have documented problems of blood pressure, pulse rate, vaginal bleeding, lochia or breastfeeding. | Output | Yes | No | No | No | No | Yes | No | Yes |
|  |  | 4 | The proportion of all newborns in the health facility who received a full clinical examination before discharge. | Output | No | No | No | No | No | No | No | No |
|  |  | 5 | The proportion of all healthy mothers and newborns who received care for at least 24 h after an uncomplicated vaginal birth in a health facility. | Output | Yes | No | Yes | No | No | No | No | No |
|  |  | 6 | The proportion of all newborns on postnatal care wards or areas in the health facility for whom there is documented information on the newborn body temperature, respiratory rate, feeding behaviour and the absence or presence of danger signs. | Output | Yes | No | No | Yes | No | No | Yes | No |
|  |  | 7 | The proportion of all healthy mothers on postnatal wards or areas in the health facility who received breastfeeding counselling and support from a skilled health care provider. | Output | Yes | No | Yes | No | No | No | No | No |
|  |  | 8 | The proportion of all women who gave birth in the health facility who were allowed to room-in with their newborn 24 h a day. | Output | Yes | No | No | No | No | Yes | No | No |
|  |  | 9 | The proportion of all postpartum women in the health facility who were offered counselling on birth spacing and family planning methods before discharge. | Output | Yes | No | No | Yes | No | No | Yes | No |
|  |  | 1 | The proportion of all newborns in the health facility who were exclusively breastfed at the time of discharge from hospital. | Outcome | Yes | Yes | N/A | N/A | N/A | N/A | N/A | N/A |
|  |  | 2 | The proportion of all postpartum women in the health facility who received contraception counselling on birth spacing and family planning before discharge. | Outcome | Yes | Yes | N/A | N/A | N/A | N/A | N/A | N/A |
| 1.2 | Women with pre-eclampsia or eclampsia promptly receive appropriate interventions. | 1 | The health facility has supplies of oral and intravenous antihypertensive agents and magnesium sulfate available in sufficient quantities at all times in the antenatal, labour and childbirth areas of the maternity unit. | Input | No | No | No | No | No | No | No | No |
|  |  | 2 | The health facility has written, up-to-date clinical protocols on the management of pre-eclampsia that are available in the labour, childbirth and postnatal areas of the maternity unit and are consistent with WHO guidelines. | Input | Yes | No | No | No | No | No | No | Yes |
|  |  | 3 | Health-care staff in the maternity unit receive in-service training and regular refresher sessions in the use of antihypertensive agents, intravenous infusion and magnesium sulfate for treating pre- eclampsia and eclampsia at least once every 12 months. | Input | Yes | No | No | Yes | No | No | Yes | Yes |
|  |  | 1 | The proportion of all women with severe pre-eclampsia or eclampsia in the health facility who received the full dose of magnesium sulfate. | Output | No | No | No | No | No | No | No | No |
|  |  | 2 | The proportion of all women with severe pregnancy-induced hypertension in the health facility who received the recommended antihypertensives. | Output | No | No | No | No | No | No | No | No |
|  |  | 3 | The proportion of all women with pre-eclampsia in the health facility whose condition progressed to eclampsia. | Output | Yes | No | No | No | No | No | No | Yes |
|  |  | 1 | The proportion of all women with pre-eclampsia or eclampsia in the health facility who died as a result of pre-eclampsia or eclampsia. | Outcome | Yes | Yes | N/A | N/A | N/A | N/A | N/A | N/A |
|  |  | 2 | The proportion of all women with pre-eclampsia or eclampsia who arrived at the health facility with a live foetus whose baby died in the perinatal period (stillbirths or early neonatal deaths). | Outcome | Yes | Yes | N/A | N/A | N/A | N/A | N/A | N/A |
|  |  | 3 | The proportion of all women with pre-eclampsia or eclampsia in the health facility who experienced maternal near-misses due to pre-eclampsia or eclampsia. | Outcome | Yes | Yes | N/A | N/A | N/A | N/A | N/A | N/A |
| 1.3 | Women with post-partum haemorrhage promptly receive appropriate interventions. | 1 | The health facility has written, up-to-date clinical protocols for post-partum haemorrhage management that are available in the childbirth and postnatal care areas and are consistent with WHO guidelines. | Input | No | No | No | No | No | No | No | No |
|  |  | 2 | The health facility has uterotonic drugs and supplies for intravenous fluid and blood administration (syringes, needles, intravenous cannulas, intravenous fluid solutions, blood) available in sufficient quantities at all times in the childbirth and postnatal care areas. | Input | No | No | No | No | No | No | No | No |
|  |  | 3 | A functional blood transfusion service is available in the health facility at all times. | Input | No | No | No | No | No | No | No | No |
|  |  | 4 | Health-care staff in the labour, childbirth and postnatal care areas of the maternity unit receive in-service training and regular refresher sessions in management of post-partum haemorrhage at least once every 12 months. | Input | Yes | No | No | Yes | No | No | Yes | Yes |
|  |  | 1 | The proportion of all women with post-partum haemorrhage in the health facility who received therapeutic uterotonic drugs. | Output | No | No | No | No | No | No | No | No |
|  |  | 2 | The proportion of all women in the health facility with post-partum haemorrhage due to a retained placenta for whom manual removal of the placenta was performed by a skilled birth attendant. | Output | No | No | No | No | No | No | No | No |
|  |  | 1 | The proportion of all women who had post-partum haemorrhage in the health facility who died as a result of post-partum haemorrhage. | Outcome | Yes | Yes | N/A | N/A | N/A | N/A | N/A | N/A |
|  |  | 2 | The proportion of all women who gave birth in the health facility by caesarean section who received a blood transfusion. | Outcome | Yes | Yes | N/A | N/A | N/A | N/A | N/A | N/A |
|  |  | 3 | The proportion of all women who gave birth vaginally in the health facility who received a blood transfusion. | Outcome | Yes | Yes | N/A | N/A | N/A | N/A | N/A | N/A |
|  |  | 4 | The proportion of all women who gave birth in the health facility who had severe post-partum haemorrhage (abnormal bleeding of >1000 mL or any bleeding with hypotension or requiring blood transfusion). | Outcome | Yes | Yes | N/A | N/A | N/A | N/A | N/A | N/A |
|  |  | 5 | The proportion of all women who had post-partum haemorrhage in the health facility who experienced maternal near-misses due to post-partum haemorrhage. | Outcome | Yes | Yes | N/A | N/A | N/A | N/A | N/A | N/A |
| 1.4 | Women whose progress in labour is delayed or whose labour is obstructed receive appropriate interventions, according to WHO guidelines. | 1 | The health facility has written, up-to-date clinical protocols for preventing and managing prolonged labour, which are available in the labour and childbirth areas and are consistent with WHO guidelines. | Input | No | No | No | No | No | No | No | No |
|  |  | 2 | The health facility has the essential supplies and equipment for vacuum or forceps-assisted delivery, including newborn resuscitation equipment, available in sufficient quantities at all times in the childbirth area of the maternity unit. | Input | No | No | No | No | No | No | No | No |
|  |  | 3 | The health facility has an adequately equipped operating theatre close to the childbirth area of the maternity unit. | Input | Yes | No | Yes | No | No | No | No | No |
|  |  | 4 | The health facility has an adequate number of staff skilled in performing caesarean section, 24 h a day. | Input | No | No | No | No | No | No | No | No |
|  |  | 5 | Health-care staff in the labour and childbirth areas of the maternity unit receive in-service training and regular refresher sessions (every 6 months) in managing prolonged and obstructed labour. | Input | Yes | No | No | Yes | No | No | Yes | Yes |
|  |  | 1 | The proportion of all nulliparous women in the health facility with a singleton cephalic foetus at ≥ 37 weeks’ gestation who underwent caesarean section during spontaneous labour (Robson group 1). | Output | No | No | No | No | No | No | No | No |
|  |  | 2 | The proportion of all women in the health facility with prolonged and/or obstructed labour who gave birth by caesarean section. | Output | No | No | No | No | No | No | No | No |
|  |  | 3 | The proportion of all women who gave birth in the health facility who underwent instrumental vaginal birth for delayed second stage of labour. | Output | No | No | No | No | No | No | No | No |
|  |  | 4 | The proportion of women with prolonged or obstructed labour who underwent emergency caesarean section within 30 min of the decision to perform caesarean section. | Output | No | No | No | No | No | No | No | No |
|  |  | 5 | The proportion of all women in the health facility with confirmed delay in progress of the first stage of labour who received oxytocin for augmentation. | Output | No | No | No | No | No | No | No | No |
|  |  | 6 | The proportion of all women who gave birth in the health facility whose active phase of first stage of labour exceeded 12 h. | Output | Yes | No | No | No | No | No | No | Yes |
|  |  | 7 | The proportion of all women who gave birth in the health facility who had a prolonged second stage of labour. | Output | Yes | No | No | No | No | No | No | Yes |
|  |  | 1 | The proportion of all women who gave birth in the health facility whose uterus ruptured during labour. | Outcome | Yes | Yes | N/A | N/A | N/A | N/A | N/A | N/A |
|  |  | 2 | The proportion of all perinatal deaths that occurred in the health facility after prolonged or obstructed labour. | Outcome | Yes | Yes | N/A | N/A | N/A | N/A | N/A | N/A |
|  |  | 3 | The proportion of all newborns born in the health facility who had birth injuries (brachial palsy, fractures, cephalohematoma). | Outcome | Yes | Yes | N/A | N/A | N/A | N/A | N/A | N/A |
|  |  | 4 | The proportion of women with prolonged and/or obstructed labour in the health facility who experienced maternal near-misses due to prolonged and/or obstructed labour. | Outcome | Yes | Yes | N/A | N/A | N/A | N/A | N/A | N/A |
| 1.5 | Newborns who are not breathing spontaneously receive appropriate stimulation and resuscitation with a bag-and-mask within 1 min of birth, according to WHO guidelines. | 1 | The health facility has a suction device, at least two sizes of neonatal mask and a self-inflating bag in the childbirth and neonatal areas of the maternity unit. | Input | No | No | No | No | No | No | No | No |
|  |  | 2 | The health facility has written, up-to-date clinical protocols for managing newborns who are not breathing spontaneously in the childbirth areas of the maternity unit that are consistent with WHO guidelines. | Input | No | No | No | No | No | No | No | No |
|  |  | 3 | All health-care workers providing care for pregnant and postpartum women and newborns in the health facility are skilled in basic newborn resuscitation, as demonstrated by simulating positive- pressure ventilation with a bag-and-mask on a manikin. | Input | Yes | No | No | Yes | No | No | Yes | No |
|  |  | 4 | Health care staff in the childbirth and neonatal areas of the maternity unit receive in-service training and regular refresher sessions in basic newborn resuscitation at least once every 12 months. | Input | Yes | No | No | Yes | No | No | Yes | Yes |
|  |  | 5 | Health care staff in the childbirth and neonatal areas of the maternity unit receive monthly drills or simulation exercises and supportive supervision in basic newborn resuscitation. | Input | Yes | No | No | Yes | No | No | Yes | Yes |
|  |  | 1 | The proportion of all newborns who were not breathing spontaneously after additional stimulation at the health facility who were resuscitated with a bag-and-mask. | Output | Yes | No | No | No | No | No | No | Yes |
|  |  | 2 | The proportion of all newborns who were not breathing spontaneously after additional stimulation at the health facility who were resuscitated with a bag-and-mask within 1 min of birth. | Output | Yes | No | No | No | No | No | No | Yes |
|  |  | 1 | The proportion of all live babies born at term (≥37weeks) with no major congenital malformations in the health facility who died within 7 days of birth (early neonatal mortality). | Outcome | Yes | Yes | N/A | N/A | N/A | N/A | N/A | N/A |
|  |  | 2 | The proportion of all live babies born at term (≥ 37 weeks) in the health facility who were not breathing spontaneously but were breathing spontaneously 5 min after resuscitation. | Outcome | Yes | Yes | N/A | N/A | N/A | N/A | N/A | N/A |
| 1.6a | Women in preterm labour receive appropriate interventions for both themselves and their babies, according to WHO guidelines. | 1 | The health facility has written, up-to-date clinical protocols for management of preterm labour in the childbirth areas of the maternity unit that are consistent with WHO guidelines. | Input | No | No | No | No | No | No | No | No |
|  |  | 2 | The health facility has supplies of antenatal corticosteroids (dexamethasone or betamethasone), antibiotics and magnesium sulfate available in sufficient quantities at all times to manage preterm birth in accordance with WHO guidelines. | Input | No | No | No | No | No | No | No | No |
|  |  | 3 | Health-care staff in the maternity unit receive in-service training and regular refresher sessions in the management of preterm labour at least once every 12 months. | Input | Yes | No | No | Yes | No | No | Yes | Yes |
|  |  | 4 | Health-care staff in the maternity unit receive monthly drills or simulation exercises and supportive supervision in the management of preterm labour. | Input | Yes | No | No | Yes | No | No | Yes | Yes |
|  |  | 1 | The proportion of all preterm newborns born between 24 and 34 weeks of gestation in the health facility whose mothers received at least one dose of antenatal corticosteroids when indicated. | Output | No | No | No | No | No | No | No | No |
|  |  | 2 | The proportion of all preterm newborns born before 32 weeks of gestation in the health facility whose mothers received magnesium sulfate to protect their baby from foetal neurological complications. | Output | Yes | No | No | No | No | No | No | Yes |
|  |  | 3 | The proportion of all women with preterm pre-labour rupture of membranes who gave birth in the health facility who received prophylactic antibiotics. | Output | Yes | No | No | No | No | No | No | Yes |
|  |  | 1 | The proportion of all women with preterm pre-labour rupture of membranes who gave birth in the health facility who received prophylactic antibiotics. | Outcome | Yes | Yes | N/A | N/A | N/A | N/A | N/A | N/A |
|  |  | 2 | The proportion of all babies at risk for birth in the health facility at 24 to < 34 weeks of gestation who were exposed to antenatal corticosteroids. | Outcome | Yes | Yes | N/A | N/A | N/A | N/A | N/A | N/A |
|  |  | 3 | The proportion of all babies at risk for birth in the health facility at≥ 34 weeks of gestation who were exposed to antenatal corticosteroids. | Outcome | Yes | Yes | N/A | N/A | N/A | N/A | N/A | N/A |
| 1.6b | Preterm and small babies receive appropriate care, according to WHO guidelines. | 1 | The health facility has written, up-to-date clinical protocols for the care of small and preterm babies in the childbirth areas of the maternity unit that are consistent with WHO guidelines. | Input | No | No | No | No | No | No | No | No |
|  |  | 2 | The health facility has supplies and materials to provide optimal thermal care to stable and unstable preterm babies, including kangaroo mother care (support binders, baby hats, socks), clean incubators and radiant warmers. | Input | No | No | No | No | No | No | No | No |
|  |  | 3 | The health facility has the supplies and materials to provide optimal feeding to preterm babies and support for breastfeeding or alternative feeding (feeding cups and spoons, infant formula, breast pumps, milk-storage facilities, pasteurizers, milk banks if possible, nasogastric tubes, syringe drivers, intravenous fluids and tubing). | Input | Yes | No | No | No | No | No | No | Yes |
|  |  | 4 | Health care staff in the health facility who work with pregnant and post-partum women and newborns receive in-service training and regular refresher sessions in appropriate care of preterm and low- birth-weight babies at least once every 12 months. | Input | Yes | No | No | Yes | No | No | Yes | Yes |
|  |  | 1 | The proportion of all low-birth-weight newborns born in the health facility with a birthweight ≤ 2000 g who received near-continuous kangaroo mother care in the first week of life. | Output | No | No | No | No | No | No | No | No |
|  |  | 2 | The proportion of all unstable low-birth-weight newborns weighing ≤ 2000 g who cannot receive kangaroo mother care in the health facility who were cared for in a thermo-neutral environment, either under radiant warmers or in incubators, as appropriate. | Output | Yes | No | No | No | No | No | No | Yes |
|  |  | 3 | The proportion of all low-birth-weight newborns born in the health facility whose mothers received additional support to establish breastfeeding. | Output | Yes | No | No | No | No | No | No | Yes |
|  |  | 1 | The proportion of all preterm babies (< 28 weeks, 28–32 weeks and 32–37 weeks of gestational age) born in the health facility who died within the first 7 days of life. | Outcome | Yes | Yes | N/A | N/A | N/A | N/A | N/A | N/A |
|  |  | 2 | The proportion of all low-birth-weight newborns born in the health facility who were exclusively fed on their mother’s milk during their stay in the health facility. | Outcome | Yes | Yes | N/A | N/A | N/A | N/A | N/A | N/A |
|  |  | 3 | The proportion of all live preterm babies born in the health facility who had severe neonatal morbidity (respiratory distress syndrome, intraventricular haemorrhage, necrotizing enterocolitis). | Outcome | Yes | Yes | N/A | N/A | N/A | N/A | N/A | N/A |
|  |  | 4 | The proportion of low-birth-weight baby deaths in the facility attributed to possible serious bacterial infection or sepsis. | Outcome | Yes | Yes | N/A | N/A | N/A | N/A | N/A | N/A |
| 1.7a | Women with or at risk for infections during labour, childbirth or the early postnatal period promptly receive appropriate interventions, according to WHO guidelines. | 1 | The health facility has supplies of oral and injectable first- and second-line antibiotics (ampicillin or penicillin and gentamicin, clindamycin, cephalosporin and metronidazole) available in sufficient quantities at all times for the expected case load. | Input | No | No | No | No | No | No | No | No |
|  |  | 2 | The health facility has written, up-to-date clinical protocols for treatment of women with, or at risk for, infections during labour, childbirth and the early postnatal period in the childbirth and postnatal care areas of the maternity unit that are consistent with WHO guidelines. | Input | No | No | No | No | No | No | No | No |
|  |  | 3 | Health care staff in the health facility who deal with pregnant and postpartum women receive in-service training and regular refresher sessions in the recognition and management of maternal peri-partum infections at least once every 12 months. | Input | Yes | No | No | Yes | No | No | Yes | Yes |
|  |  | 1 | The proportion of all women who underwent caesarean section in the health facility who received prophylactic antibiotics before caesarean section. | Output | No | No | No | No | No | No | No | No |
|  |  | 2 | The proportion of all women who gave birth in the health facility with preterm pre-labour rupture of membranes who received antibiotics. | Output | No | No | No | No | No | No | No | No |
|  |  | 3 | The proportion of all women in the health facility with third- or fourth-degree perineal tears who received antibiotics. | Output | No | No | No | No | No | No | No | No |
|  |  | 4 | The proportion of all birthing or postpartum women in the health facility with signs of infection who received injectable antibiotics. | Output | No | No | No | No | No | No | No | No |
|  |  | 5 | The proportion of all women who gave birth in the health facility who had a temperature of >38 ̊C or other signs of infection (foul-smelling or purulent lochia) after childbirth. | Output | No | No | No | No | No | No | No | No |
|  |  | 1 | The proportion of all women who underwent caesarean section in the health facility who had severe systemic infection or sepsis after the caesarean section. | Outcome | Yes | Yes | N/A | N/A | N/A | N/A | N/A | N/A |
|  |  | 2 | The proportion of all women who gave birth in the health facility who had severe systemic infection or sepsis in the postpartum period. | Outcome | Yes | Yes | N/A | N/A | N/A | N/A | N/A | N/A |
| 1.7b | Newborns with suspected infection or risk factors for infection are promptly given antibiotic treatment, according to WHO guidelines. | 1 | The health facility has supplies of injectable antibiotics (at least first- and second-line antibiotics for neonatal sepsis and meningitis) available in sufficient quantities at all times for the expected case load. | Input | No | No | No | No | No | No | No | No |
|  |  | 2 | The health facility has a written, up-to-date clinical protocol for early diagnosis and management of neonatal infection in the childbirth areas of the maternity unit that is consistent with WHO guidelines. | Input | No | No | No | No | No | No | No | No |
|  |  | 3 | Health care staff in the health facility who care for pregnant and postpartum women and newborns receive in-service training and regular refresher sessions in the recognition and management of suspected newborn infections at least once every 12 months. | Input | Yes | No | No | Yes | No | No | Yes | Yes |
|  |  | 4 | Health care staff in the health facility know the signs of newborn sepsis and how to treat it, according to WHO guidelines. | Input | Yes | No | No | No | No | Yes | No | No |
|  |  | 1 | The proportion of all newborns in the health facility with signs of infection who received injectable antibiotics. | Output | No | No | No | No | No | No | No | No |
|  |  | 2 | The proportion of all newborns of mothers with signs of infection in the health facility who received injectable antibiotics. | Output | No | No | No | No | No | No | No | No |
|  |  | 1 | The proportion of newborns treated for sepsis in the health facility who died (case fatality rate). | Outcome | Yes | Yes | N/A | N/A | N/A | N/A | N/A | N/A |
|  |  | 2 | The proportion of all neonatal deaths in the health facility that were due to sepsis. | Outcome | Yes | Yes | N/A | N/A | N/A | N/A | N/A | N/A |
|  |  | 3 | The proportion of all severe neonatal morbidity in the health facility that was due to neonatal sepsis. | Outcome | Yes | Yes | N/A | N/A | N/A | N/A | N/A | N/A |
| 1.8 | All women and newborns receive care that includes standard precautions for preventing hospital-acquired infections. | 1 | The health facility has a reliable water source on site and soap and towels (preferably disposable) or alcohol-based hand rub for hand hygiene. | Input | No | No | No | No | No | No | No | No |
|  |  | 2 | The health facility ensures safe handling, storage and final disposal of infectious waste. | Input | No | No | No | No | No | No | No | No |
|  |  | 3 | The health facility ensures safe handling, storage (puncture resistant) and final disposal of sharps waste. | Input | No | No | No | No | No | No | No | No |
|  |  | 4 | The health facility has appropriate sterilizing facilities and disinfectants for instruments. | Input | No | No | No | No | No | No | No | No |
|  |  | 5 | The health facility has a functioning incinerator or other appropriate method for treatment of infectious waste and used instruments. | Input | Yes | No | No | Yes | No | No | No | No |
|  |  | 6 | The health facility has written, up-to-date guidelines for standard infection control and precautions for transmission. | Input | Yes | No | No | No | No | Yes | No | No |
|  |  | 7 | Health care staff in the childbirth and neonatal areas of the maternity unit receive training in standard infection control and precautions for transmission at least once every 12 months. | Input | Yes | No | Yes | Yes | No | No | No | No |
|  |  | 1 | The percentage of health care staff in the health facility who clean their hands correctly as per the WHO “5 moments for hand hygiene” audit tool. | Output | No | No | No | No | No | No | No | No |
|  |  | 2 | The proportion of newborns with suspected severe bacterial infection who received appropriate antibiotic therapy. | Output | No | No | No | No | No | No | No | No |
|  |  | 3 | Safe management of health care waste, from the point of generation to the point of disposal. | Output | Yes | No | No | Yes | No | No | No | No |
|  |  | 4 | The percentage of staff members in the health facility who meet biosafety standards when administering parenteral drugs. | Output | Yes | No | No | No | No | No | No | Yes |
|  |  | 1 | The proportion of all women who gave birth in the health facility who had a severe systemic infection or sepsis in the postnatal period, including at readmission after delivery in the facility. | Outcome | Yes | Yes | N/A | N/A | N/A | N/A | N/A | N/A |
|  |  | 2 | The proportion of all women who gave birth in the health facility who had proven hospital-acquired infections. | Outcome | Yes | Yes | N/A | N/A | N/A | N/A | N/A | N/A |
|  |  | 3 | The proportion of all neonates born in the health facility who had hospital-acquired infections. | Outcome | Yes | Yes | N/A | N/A | N/A | N/A | N/A | N/A |
| 1.9 | No woman or newborn is subjected to unnecessary or harmful practices during labour, childbirth and the early postnatal period. | 1 | The health facility has written, up-to-date guidance on harmful practices and unnecessary interventions during labour, childbirth and the early postnatal period. | Input | No | No | No | No | No | No | No | No |
|  |  | 2 | The health facility does not display infant formula or bottles and teats, including on posters or placards. | Input | No | No | No | No | No | No | No | No |
|  |  | 3 | The health facility does not give newborns food or drink other than breast milk, unless medically indicated, and does not give pacifiers (also called “dummies” or “soothers”) to breastfeeding babies. | Input | Yes | No | No | No | No | No | No | Yes |
|  |  | 4 | Health care staff in the facility receive in-service training and regular refresher sessions on harmful practices and unnecessary interventions at least once every 12 months. | Input | Yes | No | No | Yes | No | No | Yes | Yes |
|  |  | 5 | The health facility does not promote infant formula on the wards, and samples are not distributed to mothers or staff. | Input | Yes | No | No | No | No | No | No | Yes |
|  |  | 6 | Health-care staff in the health facility receive monthly supportive supervision and mentoring on harmful practices and unnecessary interventions. | Input | Yes | No | No | Yes | No | No | Yes | Yes |
|  |  | 1 | The proportion of all uncomplicated, spontaneous vaginal births in the health facility in which an episiotomy was performed. | Output | No | No | No | No | No | No | No | No |
|  |  | 2 | The proportion of women undergoing caesarean section in the health facility according to Robson classification groups. | Output | No | No | No | No | No | No | No | No |
|  |  | 3 | The proportion of all women who gave birth in the health facility who received augmentation of labour with no indication of delay in progress of labour. | Output | Yes | No | No | No | No | No | No | Yes |
|  |  | 4 | The proportion of all babies born in the health facility who received early bathing and removal of the vernix within 6 h of birth. | Output | Yes | No | No | No | No | No | No | Yes |
|  |  | 5 | The proportion of all women who gave birth in the health facility who received routine pubic or perineal shaving before a vaginal birth. | Output | Yes | No | No | No | No | No | No | Yes |
|  |  | 6 | The proportion of all babies born through clear amniotic fluid in the health facility who received routine suctioning. | Output | Yes | No | No | No | No | No | No | Yes |
|  |  | 7 | The proportion of all women who gave birth in the health facility who received routine enemas at any time before vaginal birth. | Output | Yes | No | No | No | No | No | No | Yes |
| 2.1 | Every woman and newborn has a complete, accurate, standardized medical record during labour, childbirth and the early postnatal period. | 1 | The health facility has registers, data collection forms, clinical and observation charts in place at all time for routine recording and monitoring of all care processes for women and newborns. | Input | No | No | No | No | No | No | No | No |
|  |  | 2 | The health facility has a birth and death registration system in place that is linked to the national vital registration system at all times. | Input | No | No | No | No | No | No | No | No |
|  |  | 3 | The health facility has a system for classifying diseases and birth outcomes, including death, which is aligned with the ICD. | Input | Yes | No | No | No | No | Yes | No | Yes |
|  |  | 1 | The proportion of all newborns currently in the health facility who have a patient identifier and individual clinical medical record. | Output | No | No | No | No | No | No | No | No |
|  |  | 2 | The proportion of all newborns discharged from the health facility within the past 24 h who had an accurately completed record of processes of care, treatments, outcomes and diagnoses (with ICD code). | Output | Yes | No | No | Yes | No | No | No | Yes |
|  |  | 3 | The proportion of all women discharged postpartum within the past 24 h who had an accurately completed record of processes of care, treatments, outcomes and diagnoses (with ICD code). | Output | Yes | No | No | Yes | No | No | No | Yes |
| 2.2 | Every health facility has a mechanism for data collection, analysis and feedback as part of its activities for monitoring and improving performance around the time of childbirth. | 1 | The health facility has conducted reviews of maternal and perinatal deaths and near-misses at least once a month within the past six months and has a mechanism for implementing the recommendations of reviews. | Input | No | No | No | No | No | No | No | No |
|  |  | 2 | The health facility has standard operating procedures and protocols in place at all times for checking, validating and reporting data. | Input | Yes | No | No | No | No | Yes | No | Yes |
|  |  | 3 | The health facility has a data system for collecting and analysing relevant indicators and can produce visual outputs and timely reporting on paper or digitally at all times. | Input | Yes | No | No | Yes | No | No | No | Yes |
|  |  | 4 | Managers and health care workers in the health facility met at least once a month within the past six months to review process and outcome data. | Input | Yes | No | No | Yes | No | No | No | Yes |
|  |  | 5 | Managers and health care workers in the health facility used the recommendations in reviews of data for decision-making and for mentoring improved performance within the past six months. | Input | Yes | No | No | Yes | No | No | No | Yes |
|  |  | 1 | The proportion of all perinatal deaths occurring in the health facility that were reviewed with standard audit tools. | Output | No | No | No | No | No | No | No | No |
|  |  | 2 | The proportion of all maternal deaths and near-misses occurring in the health facility that were reviewed with standard audit tools. | Output | No | No | No | No | No | No | No | No |
|  |  | 3 | The proportion of all maternal deaths and near-misses occurring in the health facility that were notified. | Output | Yes | No | No | Yes | No | No | No | Yes |
|  |  | 1 | Data are collected routinely in the health facility during labour, childbirth and the postnatal period and used regularly to make decisions on quality improvement. | Outcome | Yes | Yes | N/A | N/A | N/A | N/A | N/A | N/A |
|  |  | 2 | The proportion of all recommendations in perinatal death reviews at the health facility that have been fully implemented. | Outcome | Yes | Yes | N/A | N/A | N/A | N/A | N/A | N/A |
|  |  | 3 | The proportion of all recommendations in maternal death reviews at the health facility that have been fully implemented. | Outcome | Yes | Yes | N/A | N/A | N/A | N/A | N/A | N/A |
| 3.1 | Every woman and newborn is appropriately assessed on admission, during labour and in the early postnatal period to determine whether referral is required, and the decision to refer is made without delay. | 1 | The health facility has written, up-to-date clinical protocols and guidelines for the identification, management (including pre-referral care) and referral of women with complications related to pregnancy and childbirth and in newborns. | Input | No | No | No | No | No | No | No | No |
|  |  | 2 | The health facility is equipped with appropriate medicines and medical supplies for stabilization and pre-referral treatment for referred women and newborns. | Input | No | No | No | No | No | No | No | No |
|  |  | 3 | Health care staff in the maternity unit receive in-service training and regular refresher sessions in referral protocols and guidelines at least once every 12 months. | Input | No | No | No | No | No | No | No | No |
|  |  | 1 | The proportion of women and newborns seen in the health facility in the past three months who fulfilled the facility’s criteria for referral who were actually referred. | Output | Yes | No | No | No | No | No | No | Yes |
|  |  | 2 | The proportion of all pregnant or postpartum women who could not be managed at the health facility who were transferred to a higher-level facility for childbirth or further management without delay, accompanied by a health care professional and a completed standardized referral note. | Output | Yes | No | No | No | No | No | No | Yes |
|  |  | 3 | The proportion of all sick, preterm or small newborns who could not be managed at the health facility who were transferred to an appropriate level of care within 1 h of a decision, accompanied by a health care professional and a completed standardized referral note. | Output | Yes | No | No | No | No | No | No | Yes |
|  |  | 1 | The proportion of all women admitted to the labour ward who reported receiving immediate attention on arrival at the health facility. | Outcome | Yes | Yes | N/A | N/A | N/A | N/A | N/A | N/A |
| 3.2 | For every woman and newborn who requires referral, the referral follows a pre-established plan that can be implemented without delay at any time. | 1 | The health facility has ready access to a functioning ambulance or other vehicle for emergency transport of women and newborns to referral facilities. | Input | No | No | No | No | No | No | No | No |
|  |  | 2 | There is an up-to-date list of network facilities in the same geographical area that provide referral care for women and children. | Input | No | No | No | No | No | No | No | No |
|  |  | 3 | The health facility has local arrangements to ensure that women and newborns who cannot be managed at the health facility are referred to an appropriate level of care without delay, 24 h a day, 7 days a week. | Input | Yes | No | Yes | No | No | No | No | Yes |
|  |  | 1 | The proportion of all newborns who died before or during transfer to a higher-level facility for further management. | Output | No | No | No | No | No | No | No | No |
|  |  | 2 | The proportion of all pregnant or postpartum women who died before or during transfer to a higher- level facility for childbirth for further management. | Output | No | No | No | No | No | No | No | No |
|  |  | 3 | The proportion of pregnant and postpartum women and newborns who were referred without appropriate emergency transport. | Output | Yes | No | Yes | No | No | No | No | Yes |
|  |  | 4 | The proportion of all women referred from the health facility who contributed financially to communication or to transport. | Output | Yes | No | No | No | No | Yes | No | No |
|  |  | 1 | The proportion of all women referred from the health facility who completed their referral. | Outcome | Yes | Yes | N/A | N/A | N/A | N/A | N/A | N/A |
|  |  | 2 | The proportion of all newborns referred from the health facility who completed their referral. | Outcome | Yes | Yes | N/A | N/A | N/A | N/A | N/A | N/A |
|  |  | 3 | The proportion of newborns referred from the facility who reached the referral facility without hypothermia. | Outcome | Yes | Yes | N/A | N/A | N/A | N/A | N/A | N/A |
| 3.3 | For every woman and newborn referred within or between health facilities, there is appropriate information exchange and feedback to relevant health care staff. | 1 | The health facility has a standardized referral form to document relevant demographic and clinical information, which includes clinical findings, diagnosis, pre-referral interventions or treatment given and reason for referral. | Input | No | No | No | No | No | No | No | No |
|  |  | 2 | The health facility has reliable communication methods, including a mobile phone, land line or radio, which is functioning at all times, for referrals and consultation on complicated cases. | Input | No | No | No | No | No | No | No | No |
|  |  | 3 | Evidence that the health facility has formal agreements, communication arrangements and a feedback system with referral centre(s). | Input | No | No | No | No | No | No | No | No |
|  |  | 1 | The proportion of all referred women seen at the referring facility for whom there was complete counter-referral feedback information. | Output | Yes | No | No | No | No | Yes | No | No |
|  |  | 2 | The proportion of all referred newborns seen at the referring facility for whom there was complete counter-referral feedback information. | Output | Yes | No | No | No | No | Yes | No | No |
|  |  | 3 | The proportion of all referred women and newborns seen at the referring facility who received timely care at the referral facility. | Output | Yes | No | No | No | No | No | No | Yes |
| 4.1 | All women and their families receive information about the care and have effective interactions with staff. | 1 | Easily understood health education materials, in an accessible written or pictorial format, are available in the languages of the communities served by the health facility. | Input | No | No | No | No | No | No | No | No |
|  |  | 2 | Health care staff in the maternity unit are oriented and receive in-service training at least once every 12 months to improve their interpersonal communication and counselling skills and cultural competence. | Input | No | No | No | No | No | No | No | No |
|  |  | 3 | The health facility has a written, up-to-date policy that outlines clear goals, operational plans and monitoring mechanisms to promote the interpersonal communication and counselling skills of health care staff. | Input | Yes | No | Yes | No | No | No | Yes | No |
|  |  | 4 | Health care staff in the maternity unit receive supportive supervision in interpersonal communication, counselling and cultural competence every three months. | Input | Yes | No | Yes | No | No | No | Yes | No |
|  |  | 1 | The proportion of all women discharged from the labour and childbirth area of the facility who received written and verbal information and counselling on the following elements before discharge: nutrition and hygiene, birth spacing and family planning, exclusive breastfeeding and maintaining lactation, keeping their baby warm and clean, communication and play with the baby, danger signs for the mother and newborn and where to go in case of complications. | Output | No | No | No | No | No | No | No | No |
|  |  | 2 | The proportion of all women who gave birth in the health facility who reported that they were given the opportunity to discuss their concerns and preferences. | Output | No | No | No | No | No | No | No | No |
|  |  | 3 | The proportion of health care staff in the health facility who demonstrated the following skills: active listening, asking questions, responding to questions, verifying the understanding of women and their families and supporting women in problem-solving. | Output | Yes | No | Yes | No | No | No | Yes | No |
|  |  | 1 | The proportion of all women who gave birth in the health facility who felt they were adequately informed by the care provider(s) about the examinations, actions and decisions taken for their care | Outcome | Yes | Yes | N/A | N/A | N/A | N/A | N/A | N/A |
|  |  | 2 | The proportion of all women who gave birth in the health facility who reported that their needs and preferences were taken into account during labour, childbirth and postnatal care. | Outcome | Yes | Yes | N/A | N/A | N/A | N/A | N/A | N/A |
|  |  | 3 | The proportion of all women who gave birth in the health facility who expressed satisfaction with the health services. | Outcome | Yes | Yes | N/A | N/A | N/A | N/A | N/A | N/A |
|  |  | 4 | The proportion of all women who gave birth in the health facility who reported that they were satisfied with the health education and information they received from care providers. | Outcome | Yes | Yes | N/A | N/A | N/A | N/A | N/A | N/A |
| 4.2 | All women and their families experience coordinated care, with clear, accurate information exchange between relevant health and social care professionals | 1 | The health facility has a standard form for clinical progress notes and monitoring events during labour (partograph), birth and after birth to facilitate written hand-over. | Input | No | No | No | No | No | No | No | No |
|  |  | 2 | The health facility has written, up-to-date protocols for verbal and written hand-over of women and newborns at shift changes, during intra-facility transfer, on referral to other facilities and at discharge. | Input | No | No | No | No | No | No | No | No |
|  |  | 3 | Health-care staff in the maternity unit are oriented and receive in-service training and regular refresher sessions at least once every 12 months in the clinical hand-over policy and communication of important information for hand-over, referral or discharge. | Input | Yes | No | Yes | No | No | No | Yes | No |
|  |  | 4 | The health facility has a functioning, reliable communication system for information exchange among relevant service providers. | Input | Yes | No | No | No | No | No | Yes | No |
|  |  | 1 | The proportion of women attended during labour and childbirth for whom a partograph has been completed. | Output | No | No | No | No | No | No | No | No |
|  |  | 2 | The proportion of all women who gave birth in the health facility who reported that health care staff introduced themselves and showed good knowledge of the women’s history and the care that had been given to date. | Output | Yes | No | Yes | No | No | No | Yes | Yes |
|  |  | 1 | The proportion of all women who gave birth in the health facility who expressed satisfaction with the health services. | Outcome | Yes | Yes | N/A | N/A | N/A | N/A | N/A | N/A |
|  |  | 2 | The proportion of health care staff, by cadre, and social professionals who were satisfied with the communication during clinical hand-over among members of the health care team in the health facility. | Outcome | Yes | Yes | N/A | N/A | N/A | N/A | N/A | N/A |
| 5.1 | All women and newborns have privacy around the time of labour and childbirth, and their confidentiality is respected. | 1 | The physical environment of the health facility allows privacy and the provision of respectful, confidential care, including the availability of curtains, screens, partitions and sufficient bed capacity. | Input | No | No | No | No | No | No | No | No |
|  |  | 2 | The health facility has written, up-to-date protocols to ensure privacy and confidentiality for all women and newborns in all aspects of care. | Input | No | No | No | No | No | No | No | No |
|  |  | 3 | The health facility has accountability mechanisms for redress in the event of violations of privacy, confidentiality or consent. | Input | Yes | No | No | Yes | No | No | Yes | No |
|  |  | 1 | The proportion of procedures in the health facility that require written consent for which there is an associated record of the woman’s consent. | Output | No | No | No | No | No | No | No | No |
|  |  | 2 | The proportion of all women undergoing examinations or procedures in the health facility who reported that their permission was sought before the examination or procedures were performed. | Output | Yes | No | Yes | No | No | Yes | No | No |
|  |  | 1 | The proportion of all women who gave birth in the health facility who were satisfied with the degree of privacy during their stay in the labour and childbirth areas. | Outcome | Yes | Yes | N/A | N/A | N/A | N/A | N/A | N/A |
|  |  | 2 | The proportion of all women examined and treated in the health facility who expressed satisfaction with the degree of privacy during examinations and treatment. | Outcome | Yes | Yes | N/A | N/A | N/A | N/A | N/A | N/A |
|  |  | 3 | The proportion of all women who gave birth in the health facility who expressed satisfaction with the health services. | Outcome | Yes | Yes | N/A | N/A | N/A | N/A | N/A | N/A |
| 5.2 | No woman or newborn is subjected to mistreatment, such as physical, sexual or verbal abuse, discrimination, neglect, detainment, extortion or denial of services. | 1 | The health facility has written, up-to-date, zero-tolerance non-discriminatory policies with regard to mistreatment of women and newborns. | Input | No | No | No | No | No | No | No | No |
|  |  | 2 | The health facility has a system whereby the mothers of small, sick newborns can be close to and nurse their babies. | Input | No | No | No | No | No | No | No | No |
|  |  | 3 | The fee structures for maternity and newborn care are equitable, affordable and clearly displayed. | Input | No | No | No | No | No | No | No | No |
|  |  | 4 | The health facility has written accountability mechanisms for redress in the event of mistreatment. | Input | Yes | No | Yes | No | No | Yes | No | No |
|  |  | 5 | The health facility has a written, up-to-date policy and protocols that outline women’s and families’ right to make a complaint about the care received and has an easily accessible mechanism (e.g. a box) for handing in complaints. | Input | Yes | No | Yes | No | No | Yes | No | No |
|  |  | 6 | Health care staff in the maternity unit receive in-service training and supportive supervision in respecting the rights of mothers and newborns, respectful care and accountability mechanisms. Orientation is provided for new staff. | Input | Yes | No | No | Yes | No | No | Yes | Yes |
|  |  | 7 | The health facility policy for payment specifically precludes detention of a woman or baby for non- payment. | Input | Yes | No | No | Yes | No | Yes | Yes | No |
|  |  | 8 | The health facility has a complaints box, which is easily accessible to women and their families, is periodically emptied and the contents reviewed. | Input | Yes | No | Yes | Yes | No | Yes | No | No |
|  |  | 1 | The proportion of all women who gave birth in the health facility who reported physical, verbal or sexual abuse, to themselves or their newborns, during labour or childbirth or after birth. | Output | Yes | No | No | Yes | No | Yes | Yes | No |
|  |  | 2 | The proportion of women who gave birth in the health facility who were satisfied that the facility met their religious and cultural needs. | Output | Yes | No | No | Yes | No | Yes | Yes | No |
|  |  | 3 | The proportion of women who attended the health facility who were refused care because of their inability to pay. | Output | Yes | No | No | No | No | Yes | Yes | No |
|  |  | 4 | The proportion of complaints received about respect and preservation of the dignity of women and their families. | Output | Yes | No | No | Yes | No | Yes | Yes | No |
|  |  | 5 | The proportion of women who gave birth in the health facility who were aware of the existence and location of a complaints box. | Output | Yes | No | No | Yes | No | Yes | Yes | No |
|  |  | 1 | The proportion of all women who gave birth in the health facility who expressed satisfaction with the health services. | Outcome | Yes | Yes | N/A | N/A | N/A | N/A | N/A | N/A |
|  |  | 2 | The proportion of all women who gave birth in the health facility who reported having been treated with respect and their dignity preserved. | Outcome | Yes | Yes | N/A | N/A | N/A | N/A | N/A | N/A |
|  |  | 3 | The proportion of all women in the health facility who made a complaint whose complaints were acted upon without repercussions. | Outcome | Yes | Yes | N/A | N/A | N/A | N/A | N/A | N/A |
| 5.3 | All women can make informed choices about the services they receive, and the reasons for interventions or outcomes are clearly explained. | 1 | The health facility has a written, up-to-date policy for obtaining informed consent from women before examinations and procedures. | Input | No | No | No | No | No | No | No | No |
|  |  | 2 | The health facility has a standard informed consent form that helps health care staff to provide easily understandable information to women in order to obtain their fully informed consent. | Input | No | No | No | No | No | No | No | No |
|  |  | 3 | Health care staff in the health facility receive in-service training and supportive supervision in effective informed consent procedures and in women’s right to choose care at childbirth. Orientation is provided for new staff. | Input | No | No | No | No | No | No | No | No |
|  |  | 4 | The health facility has written accountability mechanisms for redress in the event that women are denied informed choice, and the mechanism is displayed. | Input | Yes | No | No | Yes | No | No | Yes | Yes |
|  |  | 1 | The proportion of procedures in the health facility that require written consent for which there is an associated record of consent signed by the woman or a family member. | Output | No | No | No | No | No | No | No | No |
|  |  | 2 | The proportion of all women who gave birth in the health facility who felt adequately informed by health care staff regarding decisions taken about their care. | Output | Yes | No | No | Yes | No | Yes | Yes | No |
|  |  | 3 | The proportion of women who received care in the health facility who were aware that they had the right to accept or refuse treatment. | Output | Yes | No | No | Yes | No | Yes | Yes | No |
|  |  | 1 | The proportion of women who gave birth in the health facility by caesarean section who were aware of the reason for the caesarean section. | Outcome | Yes | Yes | N/A | N/A | N/A | N/A | N/A | N/A |
|  |  | 2 | The proportion of all women who gave birth in the health facility who expressed satisfaction with the health services. | Outcome | Yes | Yes | N/A | N/A | N/A | N/A | N/A | N/A |
|  |  | 3 | The proportion of women who gave birth in the health facility who felt they had shared decisions about their labour, birth and postnatal care. | Outcome | Yes | Yes | N/A | N/A | N/A | N/A | N/A | N/A |
| 6.1 | Every woman is offered the option to experience labour and childbirth with the companion of her choice. | 1 | The labour and childbirth areas are organized in such a way as to allow a physical private space for the woman and her companion at the time of birth. | Input | No | No | No | No | No | No | No | No |
|  |  | 2 | The health facility has a written, up-to-date protocol, which is explained to women and their families, to encourage all women to have at least one person of their choice, as culturally appropriate, with them during labour, childbirth and the immediate postnatal period. | Input | No | No | No | No | No | No | No | No |
|  |  | 3 | Health care staff in the health facility are oriented and receive in-service refresher training sessions at least once every 12 months on the evidence for and positive impact of the presence of a chosen companion during labour and birth. | Input | Yes | No | No | Yes | No | No | Yes | Yes |
|  |  | 4 | Orientation sessions and information (written or pictorial) are available to orient the companion on his or her role in supporting the woman during labour and birth. | Input | Yes | No | No | Yes | No | Yes | Yes | Yes |
|  |  | 1 | The proportion of all women who gave birth in the health facility who had a companion of their choice during labour and childbirth. | Output | No | No | No | No | No | No | No | No |
|  |  | 2 | The proportion of all companions who were satisfied with the orientation given on their role during labour and childbirth. | Output | Yes | No | No | No | No | No | Yes | No |
|  |  | 1 | The proportion of all women who gave birth in the health facility who expressed satisfaction with the health services. | Outcome | Yes | Yes | N/A | N/A | N/A | N/A | N/A | N/A |
| 6.2 | Every woman receives support to strengthen her capability during childbirth. | 1 | Health care staff in the labour and childbirth areas of the maternity unit were oriented in non- pharmacological and pharmacological pain relief and received in-service training or sessions at least once in the preceding 12 months. | Input | No | No | No | No | No | No | No | No |
|  |  | 2 | The health facility has a written, up-to-date protocol, which is explained to women and their families, to minimize unnecessary interventions, support normal labour and strengthen the woman’s capability, so that she feels in control of her childbirth experience. | Input | Yes | No | No | No | No | Yes | Yes | No |
|  |  | 3 | Health care staff in the labour and childbirth areas of the maternity unit were oriented and received in-service training or refresher sessions at least once in the preceding 12 months to strengthen their interpersonal and cultural competence in providing emotional support. | Input | Yes | No | No | Yes | No | No | Yes | Yes |
|  |  | 4 | The health facility has a referral mechanism for women and families with complex emotional needs, and refers them for specialist care. | Input | Yes | No | No | No | No | Yes | Yes | No |
|  |  | 1 | The proportion of all women who gave birth in the health facility who did so in the labour position of their choice. | Output | Yes | No | No | No | No | Yes | Yes | No |
|  |  | 2 | The proportion of all women undergoing bereavement or an adverse outcome who received additional emotional support from health facility staff. | Output | Yes | No | No | No | No | No | Yes | No |
|  |  | 3 | The proportion of all women who gave birth in the health facility who reported having sufficient food and drink during labour. | Output | Yes | No | No | Yes | No | Yes | Yes | No |
|  |  | 4 | The proportion of all women who gave birth in the health facility who were ambulatory during the first stage of labour. | Output | Yes | No | No | No | No | No | Yes | No |
|  |  | 1 | The proportion of all women who gave birth in the health facility who expressed satisfaction with the health services. | Outcome | Yes | Yes | N/A | N/A | N/A | N/A | N/A | N/A |
|  |  | 2 | The proportion of all women who gave birth in the health facility who would recommend childbirth in that facility. | Outcome | Yes | Yes | N/A | N/A | N/A | N/A | N/A | N/A |
|  |  | 3 | The proportion of all women who gave birth in the health facility who reported a positive birth experience. | Outcome | Yes | Yes | N/A | N/A | N/A | N/A | N/A | N/A |
|  |  | 4 | The proportion of all women who gave birth in the health facility who were satisfied that their choices and preferences were respected. | Outcome | Yes | Yes | N/A | N/A | N/A | N/A | N/A | N/A |
| 7.1 | Every woman and child has access at all times to at least one skilled birth attendant and support staff for routine care and management of complications. | 1 | The health facility has skilled birth attendants available at all times, in sufficient numbers to meet the anticipated work load. | Input | No | No | No | No | No | No | No | No |
|  |  | 2 | The health facility has a written, up-to-date staffing policy, listing the numbers, types and competence of staff, that is reviewed continuously according to the work load. | Input | No | No | No | No | No | No | No | No |
|  |  | 3 | The health facility has a roster that is displayed in all areas, giving the names of staff on duty, the times of their shifts and their specific roles and responsibilities | Input | Yes | No | Yes | No | No | Yes | No | No |
|  |  | 4 | The health facility has a written, up-to-date policy on triage and waiting times for emergency and non-emergency consultations and treatment. | Input | Yes | No | No | No | No | Yes | No | No |
|  |  | 5 | The health facility has clear communication channels to reach staff on duty at all times. | Input | Yes | No | No | No | No | Yes | No | No |
|  |  | 1 | The proportion of available posts in the health facility that were filled by staff with the necessary competence for the job description to allow the facility to provide 24-h service. | Output | Yes | No | No | No | No | No | Yes | No |
|  |  | 2 | The proportion of staff who have been oriented to their functions, roles and responsibilities in the facility or unit to which they are assigned. | Output | Yes | No | No | No | No | No | Yes | No |
|  |  | 3 | The proportion of women who attended the health facility who reported receiving attention within the appropriate time for their condition as per facility policy on triage and waiting time. | Output | Yes | No | Yes | No | No | Yes | No | No |
|  |  | 1 | The proportion of women and their newborns in the health facility who were attended by a skilled birth attendant (as per the definition) during and after childbirth. | Outcome | Yes | Yes | N/A | N/A | N/A | N/A | N/A | N/A |
|  |  | 2 | The proportion of all women who gave birth at the health facility who reported having been informed about danger signs for her and her baby and emergency preparedness. | Outcome | Yes | Yes | N/A | N/A | N/A | N/A | N/A | N/A |
|  |  | 3 | The proportion of women who attended the health facility who were satisfied with the health care they received. | Outcome | Yes | Yes | N/A | N/A | N/A | N/A | N/A | N/A |
| 7.2 | The skilled birth attendants and support staff have appropriate competence and skills to meet requirements during labour, childbirth and the early postnatal period. | 1 | The health facility has a programme for continuing professional development and skills development for all skilled birth attendants and other support staff and conducts regular training. | Input | No | No | No | No | No | No | No | No |
|  |  | 2 | The health facility has standard procedures and plans for recruitment, deployment, motivation (recognition and reward scheme) and retention of all staff. | Input | No | No | No | No | No | No | No | No |
|  |  | 3 | The health facility periodically appraises all staff and has a mechanism for recognizing good performance. | Input | Yes | No | No | No | No | Yes | Yes | No |
|  |  | 4 | The health facility has sufficient numbers of educated, competent, licensed, motivated, regulated skilled birth attendants with an appropriate skills mix, working in multidisciplinary teams. | Input | Yes | No | No | No | No | Yes | Yes | No |
|  |  | 5 | The health facility provides an enabling, supportive environment for professional staff development, with regular supportive supervision and mentoring. | Input | Yes | No | No | No | No | Yes | Yes | No |
|  |  | 6 | The health facility facilitates inter-professional collaborative practice, with clear roles and responsibilities based on the professional scope of practice and care needs during labour, childbirth and the early postnatal period. | Input | Yes | No | No | No | No | Yes | Yes | No |
|  |  | 1 | The proportion of skilled birth staff at the health facility who received a written job description on deployment to the facility. | Output | No | No | No | No | No | No | No | No |
|  |  | 2 | The proportion of skilled birth attendants at the health facility who received in-service training, a refresher session or mentoring within the past 12 months. | Output | Yes | No | No | Yes | No | No | Yes | Yes |
|  |  | 3 | The number of supervisory visits to support clinical competence and performance improvement (in the past three months). | Output | Yes | No | No | Yes | No | No | Yes | Yes |
|  |  | 4 | The proportion of staff at the health facility who were assessed at least once in the preceding 12 months. | Output | Yes | No | No | Yes | No | No | Yes | Yes |
|  |  | 5 | The number of team meetings held per month to review competence and quality improvement activities. | Output | Yes | No | No | Yes | No | No | Yes | Yes |
|  |  | 6 | The number of interactions per month with professional mentors to ensure clinical competence and improve performance. | Output | Yes | No | No | Yes | No | No | Yes | Yes |
|  |  | 7 | The proportion of all staff at the health facility who were engaged in at least two active quality improvement team meetings and participated in quality improvement activities in the preceding six months. | Output | Yes | No | No | Yes | No | No | Yes | Yes |
|  |  | 1 | The proportion of all women who gave birth at the health facility who were satisfied with the care and support from facility staff. | Outcome | Yes | Yes | N/A | N/A | N/A | N/A | N/A | N/A |
|  |  | 2 | The proportion of skilled birth attendants and support staff at the health facility whose preceding performance appraisal was satisfactory. | Outcome | Yes | Yes | N/A | N/A | N/A | N/A | N/A | N/A |
|  |  | 3 | The proportion of all staff at the health facility who reported being “highly satisfied” with their job. | Outcome | Yes | Yes | N/A | N/A | N/A | N/A | N/A | N/A |
|  |  | 4 | The proportion of all staff at the health facility who could identify and report on at least one clinical improvement activity in which they were personally involved in the past six months. | Outcome | Yes | Yes | N/A | N/A | N/A | N/A | N/A | N/A |
|  |  | 5 | The proportion of all staff at the health facility who were actively considering looking for a new job. | Outcome | Yes | Yes | N/A | N/A | N/A | N/A | N/A | N/A |
| 7.3 | The managerial and clinical leadership of every health facility is collectively responsible for creating and implementing appropriate policies and fosters an environment that supports facility staff in continuous quality improvement. | 1 | The health facility has a written, up-to-date plan for improving the quality of care and a patient safety programme. | Input | No | No | No | No | No | No | No | No |
|  |  | 2 | The health facility has a written, up-to-date leadership structure, with defined roles and responsibilities and lines of accountability for reporting. | Input | No | No | No | No | No | No | No | No |
|  |  | 3 | The health facility has a designated quality improvement team and responsible personnel. | Input | No | No | No | No | No | No | No | No |
|  |  | 4 | The health facility has a mechanism for regular collection of information on patient and provider satisfaction. | Input | Yes | No | No | No | No | Yes | No | No |
|  |  | 5 | The health facility holds at least one monthly meeting to review data, monitor quality improvement performance, make recommendations to address any identified problems, honour those who have performed well and encourage staff who are struggling to improve. | Input | Yes | No | No | Yes | No | Yes | No | No |
|  |  | 6 | All standard governing procedures (policies and protocols) are in place and accessible to all relevant staff. | Input | Yes | No | No | No | No | Yes | No | No |
|  |  | 7 | The proportion of all health facility leaders who were trained in quality improvement and leading change (use of information, enabling behaviour, continuous learning). | Input | Yes | No | No | Yes | No | No | Yes | Yes |
|  |  | 8 | The health facility holds at least two annual meetings with stakeholders (e.g. the community, service users, partners) to review its performance, identify problems and make recommendations for joint actions to improve quality. | Input | Yes | No | No | Yes | No | No | Yes | Yes |
|  |  | 9 | The proportion of all health facility leaders who were trained in leadership and management skills. | Input | Yes | No | No | Yes | No | No | Yes | Yes |
|  |  | 10 | A policy is in place for staff to provide feedback to the facility management on quality improvement and their performance. | Input | Yes | No | No | No | No | Yes | No | No |
|  |  | 1 | Health facility leaders communicated the performance of the facility through established mechanisms for monitoring (e.g. a dashboard of key metrics) to all relevant staff. | Output | Yes | No | No | No | No | Yes | No | No |
|  |  | 2 | The proportion of monthly meetings on the quality of care that were actually held in the preceding 12 months. | Output | Yes | No | No | No | No | No | Yes | No |
|  |  | 1 | The proportion of all women who gave birth at the health facility who were satisfied with the care and support from facility staff. | Outcome | Yes | Yes | N/A | N/A | N/A | N/A | N/A | N/A |
|  |  | 2 | Evidence for improved performance of the system (according to the facility dashboard). | Outcome | Yes | Yes | N/A | N/A | N/A | N/A | N/A | N/A |
| 8.1 | Water, energy, sanitation, hand hygiene and waste disposal facilities are functioning, reliable, safe and sufficient for the needs of staff, women and their families. | 1 | The health facility has a functioning source of safe water located on the premises that is adequate to meet all demands for drinking, personal hygiene, medical interventions, cleaning, laundry and cooking for use by staff, women, newborns and their families. | Input | No | No | No | No | No | No | No | No |
|  |  | 2 | The health facility has leak-proof, covered, labelled waste bins and impermeable sharps containers available in every treatment area, to allow segregation of waste into four categories: sharps, non- sharps infectious waste, general non-infectious waste (e.g. food, packaging) and anatomical waste (e.g. placenta). | Input | No | No | No | No | No | No | No | No |
|  |  | 3 | The health facility has at least one functioning hand hygiene station per 10 beds, with soap and water or alcohol-based hand rubs, in all wards. | Input | No | No | No | No | No | No | No | No |
|  |  | 4 | The health facility has energy infrastructure (e.g. solar, generator, grid) that can meet all the electricity demands of the facility and associated infrastructure at all times, with a back-up power source. | Input | No | No | No | No | No | No | No | No |
|  |  | 5 | The health facility has written, up-to-date protocols and awareness-raising materials (posters) on cleaning and disinfection, hand hygiene, operating and maintaining water, sanitation and hygiene facilities and safe waste management; these are posted in the areas in which the activities are conducted. | Input | Yes | No | No | No | No | Yes | Yes | No |
|  |  | 6 | The health facility has sanitation facilities on premises that are usable, appropriately illuminated at night, accessible to people with limited mobility and separated by gender for staff and patients; they include at least one toilet that meets the needs for menstrual hygiene management, with hand- washing stations and soap and water (at least 1 latrine per 20 users for inpatient settings). | Input | Yes | No | No | No | No | No | Yes | No |
|  |  | 7 | The health facility has sufficient trained, competent staff on site when needed, with clear descriptions of their responsibilities for cleaning, operating and maintaining water, sanitation, hygiene and health care waste facilities. | Input | Yes | No | No | Yes | No | No | Yes | Yes |
|  |  | 8 | The health facility has sufficient funds for rehabilitation, improvement and continuous operation and maintenance of water, sanitation, hygiene and health care waste services. | Input | Yes | No | No | No | No | No | Yes | Yes |
|  |  | 9 | The heath facility has a fuel management plan and a local buffer stock, supported by an adequate budget for all the fuel needs for vehicles, cooking and heating, as relevant and as required, at all times. | Input | Yes | No | No | No | No | No | Yes | Yes |
|  |  | 10 | The health facility has a preventive risk plan for managing and improving water, sanitation and hygiene services, including for infection prevention and control. | Input | Yes | No | No | No | No | No | No | Yes |
|  |  | 11 | The heath facility has an energy management plan supported by an adequate budget, maintained by appropriately trained staff and regulated by a competent authority. | Input | Yes | No | No | No | No | No | Yes | Yes |
|  |  | 1 | The proportion of women and their families who attended the health facility who were satisfied with the water, sanitation and energy services and would recommend the health facility to friends and family. | Outcome | Yes | Yes | N/A | N/A | N/A | N/A | N/A | N/A |
|  |  | 2 | The proportion of all health care staff at the health facility who were satisfied with the water, sanitation and energy services and considered that these services contribute positively to providing high-quality care. | Outcome | Yes | Yes | N/A | N/A | N/A | N/A | N/A | N/A |
|  |  | 3 | The proportion of women and their families who attended the health facility who were satisfied with the power and lighting source and would recommend the health facility to friends and family. | Outcome | Yes | Yes | N/A | N/A | N/A | N/A | N/A | N/A |
| 8.2 | Areas for labour, childbirth and postnatal care are designed, organized and maintained so that every woman and newborn can be cared for according to their needs in private, to facilitate the continuity of care. | 1 | The health facility has a dedicated area in the labour and childbirth area for resuscitation of newborns, which is adequately equipped with a table or resuscitaire, radiant warmer, light and appropriate resuscitation equipment and supplies. | Input | No | No | No | No | No | No | No | No |
|  |  | 2 | The health facility has a labour ward and an adequate number of birthing rooms or areas for the estimated number of births in the service area. | Input | No | No | No | No | No | No | No | No |
|  |  | 3 | The health facility has clean, appropriately illuminated, well-ventilated labour, childbirth and neonatal areas and surroundings that allow for privacy and are adequately equipped, regularly cleaned and maintained. | Input | No | No | No | No | No | No | No | No |
|  |  | 4 | The health facility practices and enables rooming-in for all women to allow mothers and babies to remain together 24 h a day. | Input | Yes | No | No | Yes | No | No | No | No |
|  |  | 5 | The health facility has a labour and childbirth area or room with a functional, clean and accessible bathroom or shower room and toilet for use only by women in labour. | Input | Yes | No | No | Yes | No | No | No | No |
|  |  | 6 | A facility offering surgical services has an adequately equipped operating theatre located close to and easily accessible from the labour and childbirth areas. | Input | Yes | No | No | No | No | Yes | Yes | No |
|  |  | 7 | The facility has a dedicated recovery room or area for care of women with complications. | Input | Yes | No | No | No | No | Yes | Yes | No |
|  |  | 8 | The health facility has a dedicated ward for admitting sick and unstable small babies. | Input | Yes | No | No | No | No | Yes | Yes | No |
|  |  | 1 | The proportion of all pregnant women who attended the health facility who reported that it has a clean physical environment conducive for childbirth. | Output | Yes | No | No | Yes | No | No | Yes | Yes |
|  |  | 1 | The proportion of all women who gave birth in the health facility who were satisfied with the environment of the labour and childbirth area, including the cleanliness, proximity to a toilet, general lighting, level of crowding and privacy. | Outcome | Yes | Yes | N/A | N/A | N/A | N/A | N/A | N/A |
| 8.3 | Adequate stocks of medicines, supplies and equipment are available for routine care and management of complications. | 1 | The health facility has supplies of antihypertensive agents and magnesium sulfate in sufficient quantities, available at all times, in antenatal, labour, childbirth and postnatal areas for the management of women with pre-eclampsia. | Input | No | No | No | No | No | No | No | No |
|  |  | 2 | The health facility has uterotonic drugs and supplies for intravenous infusion (syringes, needles, infusion sets, intravenous fluid solutions and blood) available in sufficient quantities at all times in the childbirth and postnatal care areas for the management of women with postpartum haemorrhage. | Input | No | No | No | No | No | No | No | No |
|  |  | 3 | The health facility has supplies of antenatal corticosteroids (dexamethasone or betamethasone), antibiotics and magnesium sulfate available in sufficient quantities at all times to manage preterm births. | Input | No | No | No | No | No | No | No | No |
|  |  | 4 | The health facility has functioning essential equipment and supplies for the detection of complications (e.g. thermometers, sphygmomanometers, foetal stethoscopes, urine dipsticks, pulse oximeter) in sufficient quantities at all times in the labour and childbirth areas of the maternity unit. | Input | Yes | No | No | No | No | Yes | Yes | No |
|  |  | 5 | The health facility has supplies of first- and second-line injectable antibiotics and other essential medicines available at all times for the management of women and newborns with, or at risk for, infections during labour, childbirth and the early postnatal period. | Input | Yes | No | No | No | No | Yes | Yes | No |
|  |  | 6 | The health facility has essential laboratory supplies and tests (blood glucose, haemoglobin or packed cell volume, blood group and cross-matching, bilirubin, urine protein, full blood count, blood culture, electrolytes, renal and liver function tests, syphilis, HIV and malaria rapid diagnostic tests) to support the management of women and newborns. | Input | Yes | No | No | No | No | Yes | Yes | No |
|  |  | 7 | The health facility has essential supplies and functioning equipment (including childbirth beds, vacuum, forceps, incubators, weighing machine, sterile gloves) available in sufficient quantities at all times in the labour and childbirth areas. | Input | Yes | No | No | No | No | Yes | Yes | No |
|  |  | 8 | The health facility has supplies and functioning equipment for the emergency care and resuscitation of women (well-stocked resuscitation trolley, suction device, pulse oximeter, airways, laryngoscope, endotracheal tubes, adult bag valve masks, infusion sets, intravenous fluids) available in sufficient quantities all times in areas designated for labour, childbirth and postnatal care. | Input | Yes | No | No | No | No | Yes | Yes | No |
|  |  | 9 | The health facility has a safe, uninterrupted oxygen source and delivery supplies (nasal prongs, catheters and masks), including nasal continuous positive airway pressure, available at all times in labour, childbirth and neonatal areas and the operating theatre (when available). | Input | Yes | No | No | No | No | Yes | Yes | No |
|  |  | 10 | The health facility has supplies and functioning equipment for emergency care and resuscitation of newborns (resuscitation table, well-stocked neonatal resuscitation trolley, warmer, suction device, pulse oximeter, laryngoscope) available all times in areas designated for labour, childbirth and neonatal care. | Input | Yes | No | No | No | No | Yes | Yes | No |
|  |  | 11 | The health facility has an on-site pharmacy and a medicine and supplies stock management system managed by a trained pharmacist or dispenser. | Input | Yes | No | No | No | No | Yes | Yes | No |
|  |  | 12 | The health facility has a dedicated budget for essential medicines, equipment (and its maintenance) and medical supplies for maternal and newborn care. | Input | Yes | No | No | No | No | Yes | Yes | No |
|  |  | 13 | The health facility has a functioning diagnostic ultrasound machine and trained health staff who can conduct a basic obstetric ultrasound examination to determine the number of fetuses present, gestational age, prenatal diagnosis of foetal anomalies or early diagnosis of placental insufficiency. | Input | Yes | No | No | No | No | Yes | Yes | No |
|  |  | 1 | Availability of essential life-saving medicines (oxytocin, magnesium sulfate, dexamethasone, vitamin K, injectable and oral amoxicillin, benzyl penicillin, gentamicin, ceftriaxone, metronidazole, antimalarial drugs, antiretroviral drugs and vaccines against tuberculosis, hepatitis B, poliomyelitis) in the past three months. | Output | Yes | No | No | No | No | Yes | Yes | No |
|  |  | 2 | The proportion of all women who had severe pre-eclampsia or eclampsia in the health facility who did not receive the full dose of magnesium sulfate because of a stock-out. | Output | Yes | No | No | No | No | No | No | Yes |
|  |  | 3 | The proportion of all women who gave birth in the health facility who purchased gloves and other necessary items. | Output | Yes | No | No | Yes | No | No | Yes | Yes |
|  |  | 1 | The proportion of all nulliparous women with a singleton cephalic foetus at ≥ 37 weeks of gestation who underwent caesarean section during spontaneous labour (Robson group 1). | Outcome | Yes | Yes | N/A | N/A | N/A | N/A | N/A | N/A |
|  |  | 2 | The proportion of unmet need for caesarean section as a result of lack of supplies or staff trained to conduct caesarean section. | Outcome | Yes | Yes | N/A | N/A | N/A | N/A | N/A | N/A |
