# Supplementary material for: Translating international guidelines for use in routine maternal and neonatal healthcare quality measurement
Source: Glob Health Action. 2020 Jul 13;13(1):1783956. doi: 10.1080/16549716.2020.1783956 (PMC7480423; doi:10.1080/16549716.2020.1783956)
Supplement: Supplemental Material [file ZGHA_A_1783956_SM0135.zip › MHQ paper_SupplementaryFileTable2_8May2020.docx]

**Supplementary Data Table 2: GRADE assessment of the quality of evidence identified in rapid review**

| **QS#** | **Quality Statement** | **Ind#** | **Indicator** | **Indicator Type** | **Sources identified in rapid review** | **[Starting score]**  **Type of evidence** | **Risk of bias** | **Imprecision** | **Inconsistency** | **Indirectness** | **GRADE score** |
| --- | --- | --- | --- | --- | --- | --- | --- | --- | --- | --- | --- |
| 1.1a | Women are assessed routinely on admission and during labour and childbirth and are given timely, appropriate care. | 1 | The health facility has the basic essential equipment and supplies for routine care and detection of complications (thermometers, sphygmomanometers, fetal stethoscopes, urine dipsticks) available in sufficient quantities at all times in the areas of the maternity unit for labour and childbirth. | Input | [1–3] | [+1] 2 cross-sectional studies and 1 qualitative study | 0 | 0 | 0 | 0 | [1] Very low |
|  |  | 2 | The health facility has written, up-to-date clinical protocols for assessing intrapartum care and action in the labour and childbirth areas of the maternity unit that are consistent with WHO guidelines. | Input |  |  |  |  |  |  | No evidence found |
|  |  | 2 | The proportion of all women who gave birth in the health facility who received oxytocin within 1 min of the birth of their baby. | Output | [4,5] | [+4] 2 systematic reviews | 0 | 0 | 0 | [-1] | [3] Moderate |
| 1.1b | Newborns receive routine care immediately after birth. | 1 | The health facility has written, up-to-date, clinical protocols for essential newborn care that are consistent with WHO guidelines and are available in the labour and childbirth areas of the maternity unit. | Input |  |  |  |  |  |  | No evidence found |
|  |  | 2 | The health facility has supplies of sterile cord ties (or clamps) and scissors (or blades), available in sufficient quantities at all times for the expected number of births. | Input | [2,6] | [+4] 1 systematic review; 1 cross-sectional study | 0 | 0 | 0 | [-1] | [3] Moderate |
|  |  | 1 | The proportion of all newborns who were breastfed within 1 h of birth. | Output | [7–9] | [+4] 1 systematic review; 1 pooled analysis of RCTs; 1 cohort study | 0 | 0 | 0 | 0 | [4] High |
|  |  | 2 | The proportion of all newborns who were kept in skin-to-skin contact (with body and head covered) with their mothers for at least 1 h after birth. | Output | [10–12] | [+4] 1 systematic review; 2 RCTs | 0 | 0 | [-1] | 0 | [3] Moderate |
|  |  | 4 | The proportion of all newborns whose umbilical cord was clamped 1–3 min after birth. | Output | [13–15] | [+4] 3 systematic reviews | 0 | 0 | 0 | [-1] | [3] Moderate |
|  |  | 5 | The proportion of all newborns who were dried immediately and thoroughly at birth. | Output | [16–18] | [+4] 1 RCT, 2 cross-sectional studies | 0 | [-1] | 0 | 0 | [3] Moderate |
| 1.1c | Mothers and newborns receive routine postnatal care. | 1 | The health facility has written, up-to-date clinical protocols for postnatal care in the maternity and/ or postnatal care areas of the maternity unit that are consistent with WHO guidelines. | Input |  |  |  |  |  |  | No evidence found |
|  |  | 2 | The health facility practises and enables rooming-in to allow mothers and babies to remain together 24 h a day. | Input | [19–22] | [+4] 1 systematic review; 1 RCT; 2 cross-sectional studies | 0 | [-1] | [-1] | 0 | [2] Low |
|  |  | 1 | The proportion of all newborns on postnatal care wards or areas in the health facility who received vitamin K and full vaccination as per national guidelines. | Output | [23,24] | [+4] 1 systematic review; 1 non-systematic review | [-1] | 0 | 0 | 0 | [3] Moderate |
|  |  | 4 | The proportion of all newborns in the health facility who received a full clinical examination before discharge. | Output | [25,26] | [+4] 1 RCT, 1 cohort study | 0 | 0 | 0 | [-1] | [3] Moderate |
| 1.2 | Women with pre-eclampsia or eclampsia promptly receive appropriate interventions. | 1 | The health facility has supplies of oral and intravenous antihypertensive agents and magnesium sulfate available in sufficient quantities at all times in the antenatal, labour and childbirth areas of the maternity unit. | Input | [27] | [+4] 1 systematic review | 0 | 0 | 0 | [-1] | [3] Moderate |
|  |  | 1 | The proportion of all women with severe pre-eclampsia or eclampsia in the health facility who received the full dose of magnesium sulfate. | Output | [28–31] | [+4] 4 systematic reviews | 0 | 0 | 0 | 0 | [4] High |
|  |  | 2 | The proportion of all women with severe pregnancy-induced hypertension in the health facility who received the recommended antihypertensives. | Output | [27] | [+4] (1 systematic review) | 0 | 0 | 0 | 0 | [4] High |
| 1.3 | Women with post-partum haemorrhage promptly receive appropriate interventions. | 1 | The health facility has written, up-to-date clinical protocols for post-partum haemorrhage management that are available in the childbirth and postnatal care areas and are consistent with WHO guidelines. | Input |  |  |  |  |  |  | No evidence found |
|  |  | 2 | The health facility has uterotonic drugs and supplies for intravenous fluid and blood administration (syringes, needles, intravenous cannulas, intravenous fluid solutions, blood) available in sufficient quantities at all times in the childbirth and postnatal care areas. | Input | [32] | [+4]  1 systematic review | 0 | 0 | 0 | [-1] | [3] Moderate |
|  |  | 3 | A functional blood transfusion service is available in the health facility at all times. | Input | [33,34] | [+4] 1 systematic review; 1 cross-sectional study | 0 | 0 | 0 | 0 | [4] High |
|  |  | 1 | The proportion of all women with post-partum haemorrhage in the health facility who received therapeutic uterotonic drugs. | Output | [35–37] | [+4] 3 systematic review | 0 | 0 | 0 | 0 | [4] High |
|  |  | 2 | The proportion of all women in the health facility with post-partum haemorrhage due to a retained placenta for whom manual removal of the placenta was performed by a skilled birth attendant. | Output | [38–40] | [+4] 2 systematic reviews; 1 non-systematic review | 0 | 0 | 0 | [-1] | [3] Moderate |
| 1.4 | Women whose progress in labour is delayed or whose labour is obstructed receive appropriate interventions, according to WHO guidelines. | 1 | The health facility has written, up-to-date clinical protocols for preventing and managing prolonged labour, which are available in the labour and childbirth areas and are consistent with WHO guidelines. | Input |  |  |  |  |  |  | No evidence found |
|  |  | 2 | The health facility has the essential supplies and equipment for vacuum or forceps-assisted delivery, including newborn resuscitation equipment, available in sufficient quantities at all times in the childbirth area of the maternity unit. | Input | [41–43] | [+4] 1 systematic review; 2 cross-sectional studies | 0 | 0 | 0 | [-1] | [3] Moderate |
|  |  | 4 | The health facility has an adequate number of staff skilled in performing caesarean section, 24 h a day. | Input | [44,45] | [+4] 1 RCT; 1 cross-sectional study | 0 | 0 | [-1] | [-1] | [2] Low |
|  |  | 1 | The proportion of all nulliparous women in the health facility with a singleton cephalic foetus at ≥ 37 weeks’ gestation who underwent caesarean section during spontaneous labour (Robson group 1). | Output | [46–49] | [+4] 3 systematic reviews, 1 cohort studies | [-1] | 0 | 0 | [-1] | [2] Low |
|  |  | 2 | The proportion of all women in the health facility with prolonged and/or obstructed labour who gave birth by caesarean section. | Output | [50] | [+4] 1 systematic review | 0 | 0 | 0 | [-1] | [3] Moderate |
|  |  | 3 | The proportion of all women who gave birth in the health facility who underwent instrumental vaginal birth for delayed second stage of labour. | Output | [42,51,52] | [+4] 2 systematic reviews, 1 cohort study | 0 | 0 | [-1] | 0 | [3] Moderate |
|  |  | 4 | The proportion of women with prolonged or obstructed labour who underwent emergency caesarean section within 30 min of the decision to perform caesarean section. | Output | [38,53] | [+4] 2 systematic reviews | 0 | 0 | [-1] | [-1] | [2] Low |
|  |  | 5 | The proportion of all women in the health facility with confirmed delay in progress of the first stage of labour who received oxytocin for augmentation. | Output | [54–56] | [+4] 2 RCTs; 1 meta-analysis | 0 | 0 | 0 | 0 | [4] High |
| 1.5 | Newborns who are not breathing spontaneously receive appropriate stimulation and resuscitation with a bag-and-mask within 1 min of birth, according to WHO guidelines. | 1 | The health facility has a suction device, at least two sizes of neonatal mask and a self-inflating bag in the childbirth and neonatal areas of the maternity unit. | Input | [57,58] | [+4] 1 systematic review; 1 cross-sectional survey | 0 | 0 | 0 | [-1] | [3] Moderate |
|  |  | 2 | The health facility has written, up-to-date clinical protocols for managing newborns who are not breathing spontaneously in the childbirth areas of the maternity unit that are consistent with WHO guidelines. | Input |  |  |  |  |  |  | No evidence found |
| 1.6a | Women in preterm labour receive appropriate interventions for both themselves and their babies, according to WHO guidelines. | 1 | The health facility has written, up-to-date clinical protocols for management of preterm labour in the childbirth areas of the maternity unit that are consistent with WHO guidelines. | Input |  |  |  |  |  |  | No evidence found |
|  |  | 2 | The health facility has supplies of antenatal corticosteroids (dexamethasone or betamethasone), antibiotics and magnesium sulfate available in sufficient quantities at all times to manage preterm birth in accordance with WHO guidelines. | Input | [59–61] | [+4] (2 systematic reviews; 1 cohort study) | 0 | 0 | 0 | [-1] | [3] Moderate |
|  |  | 1 | The proportion of all preterm newborns born between 24 and 34 weeks of gestation in the health facility whose mothers received at least one dose of antenatal corticosteroids when indicated. | Output | [59–61] | [+4] (2 systematic reviews; 1 cohort study) | [-1] | 0 | 0 | [-1] | [2] Low |
| 1.6b | Preterm and small babies receive appropriate care, according to WHO guidelines. | 1 | The health facility has written, up-to-date clinical protocols for the care of small and preterm babies in the childbirth areas of the maternity unit that are consistent with WHO guidelines. | Input |  |  |  |  |  |  | No evidence found |
|  |  | 2 | The health facility has supplies and materials to provide optimal thermal care to stable and unstable preterm babies, including kangaroo mother care (support binders, baby hats, socks), clean incubators and radiant warmers. | Input | [62–65] | [+4] 3 systematic reviews; 1 quasi-randomized study | 0 | 0 | 0 | [-1] | [3] Moderate |
|  |  | 1 | The proportion of all low-birth-weight newborns born in the health facility with a birth weight ≤ 2000 g who received near-continuous kangaroo mother care in the first week of life. | Output | [62–65] | [+4] 3 systematic reviews; 1 quasi-randomized study | 0 | 0 | 0 | [-1] | [3] Moderate |
| 1.7a | Women with or at risk for infections during labour, childbirth or the early postnatal period promptly receive appropriate interventions, according to WHO guidelines. | 1 | The health facility has supplies of oral and injectable first- and second-line antibiotics (ampicillin or penicillin and gentamicin, clindamycin, cephalosporin and metronidazole) available in sufficient quantities at all times for the expected case load. | Input | [66–74] | [+4] 9 systematic reviews | 0 | 0 | 0 | [-1] | [3] Moderate |
|  |  | 2 | The health facility has written, up-to-date clinical protocols for treatment of women with, or at risk for, infections during labour, childbirth and the early postnatal period in the childbirth and postnatal care areas of the maternity unit that are consistent with WHO guidelines. | Input |  |  |  |  |  |  | No evidence found |
|  |  | 1 | The proportion of all women who underwent caesarean section in the health facility who received prophylactic antibiotics before caesarean section. | Output | [68,69,72] | [+4] 3 systematic reviews | [-1] | 0 | 0 | [-1] | [2] Low |
|  |  | 2 | The proportion of all women who gave birth in the health facility with preterm pre-labour rupture of membranes who received antibiotics. | Output | [66,70,74] | [+4] 3 systematic reviews | 0 | 0 | [-1] | [-1] | [2] Low |
|  |  | 3 | The proportion of all women in the health facility with third- or fourth-degree perineal tears who received antibiotics. | Output | [71] | [+4] 1 systematic review | 0 | [-1] | 0 | 0 | [3] Moderate |
|  |  | 4 | The proportion of all birthing or postpartum women in the health facility with signs of infection who received injectable antibiotics. | Output | [67,73] | [+4] 2 systematic reviews | [-1] | [-1] | 0 | 0 | [2] Low |
|  |  | 5 | The proportion of all women who gave birth in the health facility who had a temperature of > 38 ˚C or other signs of infection (foul-smelling or purulent lochia) after childbirth. | Output |  |  |  |  |  |  | No evidence found |
| 1.7b | Newborns with suspected infection or risk factors for infection are promptly given antibiotic treatment, according to WHO guidelines. | 1 | The health facility has supplies of injectable antibiotics (at least first- and second-line antibiotics for neonatal sepsis and meningitis) available in sufficient quantities at all times for the expected case load. | Input | [75–77] | [+4] 3 systematic reviews | 0 | 0 | [-1] | 0 | Moderate |
|  |  | 2 | The health facility has a written, up-to-date clinical protocol for early diagnosis and management of neonatal infection in the childbirth areas of the maternity unit that is consistent with WHO guidelines. | Input |  |  |  |  |  |  | No evidence found |
|  |  | 1 | The proportion of all newborns in the health facility with signs of infection who received injectable antibiotics. | Output | [75,76] | [+4] 2 systematic reviews | 0 | 0 | [-1] | [-1] | [2] Low |
|  |  | 2 | The proportion of all newborns of mothers with signs of infection in the health facility who received injectable antibiotics. | Output | [77] | [+4] 1 systematic review | [-1] | 0 | 0 | [-1] | [2] Low |
| 1.8 | All women and newborns receive care that includes standard precautions for preventing hospital-acquired infections. | 1 | The health facility has a reliable water source on site and soap and towels (preferably disposable) or alcohol-based hand rub for hand hygiene. | Input | [78] | [+4] 1 systematic review | 0 | 0 | 0 | [-1] | [3] Moderate |
|  |  | 2 | The health facility ensures safe handling, storage and final disposal of infectious waste. | Input | [79–81] | [+4] 1 systematic review, 2 non-systematic reviews | 0 | 0 | 0 | 0 | [4] High |
|  |  | 3 | The health facility ensures safe handling, storage (puncture resistant) and final disposal of sharps waste. | Input | [82,83] | [+2] 2 cross-sectional studies | 0 | 0 | 0 | 0 | [2] Low |
|  |  | 4 | The health facility has appropriate sterilizing facilities and disinfectants for instruments. | Input | [84] | [+2] 1 non-systematic review | 0 | 0 | 0 | 0 | [2] Low |
|  |  | 1 | The percentage of health care staff in the health facility who clean their hands correctly as per the WHO “5 moments for hand hygiene” audit tool. | Output | [85–87] | [+2] 1 quasi-experimental study, 1 cohort study, 1 cross-sectional study | 0 | [-1] | 0 | 0 | [1] Very low |
|  |  | 2 | The proportion of newborns with suspected severe bacterial infection who received appropriate antibiotic therapy. | Output | [88,89] | [+4] 2 systematic reviews | 0 | 0 | 0 | 0 | [4] High |
| 1.9 | No woman or newborn is subjected to unnecessary or harmful practices during labour, childbirth and the early postnatal period. | 1 | The health facility has written, up-to-date guidance on harmful practices and unnecessary interventions during labour, childbirth and the early postnatal period. | Input |  |  |  |  |  |  | No evidence found |
|  |  | 2 | The health facility does not display infant formula or bottles and teats, including on posters or placards. | Input | [90–92] | [+2] 2 non-systematic reviews, 1 cross sectional survey | 0 | 0 | 0 | [-1] | 1 (very low) |
|  |  | 1 | The proportion of all uncomplicated, spontaneous vaginal births in the health facility in which an episiotomy was performed. | Output | [93] | [+4] 1 systematic rev | 0 | 0 | 0 | 0 | [4] High |
|  |  | 2 | The proportion of women undergoing caesarean section in the health facility according to Robson classification groups. | Output | [46–49] | [+4] 3 systematic reviews, 1 cohort studies | [-1] | 0 | 0 | 0 | [3] Moderate |
| 2.1 | Every woman and newborn has a complete, accurate, standardized medical record during labour, childbirth and the early postnatal period. | 1 | The health facility has registers, data collection forms, clinical and observation charts in place at all time for routine recording and monitoring of all care processes for women and newborns. | Input | [94–96] | [+4] 2 systematic reviews, 1 cross sectional study | 0 | 0 | [-1] | 0 | [3] Moderate |
|  |  | 2 | The health facility has a birth and death registration system in place that is linked to the national vital registration system at all times. | Input | [97,98] | [+2]  2 cross-sectional studies | 0 | 0 | 0 | [-1] | [1] Very low |
|  |  | 1 | The proportion of all newborns currently in the health facility who have a patient identifier and individual clinical medical record. | Output |  |  |  |  |  |  | No evidence found |
| 2.2 | Every health facility has a mechanism for data collection, analysis and feedback as part of its activities for monitoring and improving performance around the time of childbirth. | 1 | The health facility has conducted reviews of maternal and perinatal deaths and near-misses at least once a month within the past six months and has a mechanism for implementing the recommendations of reviews. | Input | [99,100] | [+4] 1 systematic review, 1 qualitative study | 0 | 0 | 0 | [-1] | [3] Moderate |
|  |  | 1 | The proportion of all perinatal deaths occurring in the health facility that were reviewed with standard audit tools. | Output | [99–102] | [+4] 1 systematic review, 2 cohort studies, 1 qualitative study | 0 | 0 | 0 | 0 | [4] High |
|  |  | 2 | The proportion of all maternal deaths and near-misses occurring in the health facility that were reviewed with standard audit tools. | Output | [99,100] | [+4] 1 systematic review, 1 qualitative study | 0 | 0 | 0 | 0 | [4] High |
| 3.1 | Every woman and newborn is appropriately assessed on admission, during labour and in the early postnatal period to determine whether referral is required, and the decision to refer is made without delay. | 1 | The health facility has written, up-to-date clinical protocols and guidelines for the identification, management (including pre-referral care) and referral of women with complications related to pregnancy and childbirth and in newborns. | Input |  |  |  |  |  |  | No evidence found |
|  |  | 2 | The health facility is equipped with appropriate medicines and medical supplies for stabilization and pre-referral treatment for referred women and newborns. | Input | [103–105] | [+2] 1 cohort study; 2 cross-sectional study | 0 | 0 | 0 | 0 | [2] Low |
|  |  | 3 | Health care staff in the maternity unit receive in-service training and regular refresher sessions in referral protocols and guidelines at least once every 12 months. | Input | [106] | [+2] 1 meta-analysis | 0 | 0 | 0 | [-1] | [1] Very low |
| 3.2 | For every woman and newborn who requires referral, the referral follows a pre-established plan that can be implemented without delay at any time. | 1 | The health facility has ready access to a functioning ambulance or other vehicle for emergency transport of women and newborns to referral facilities. | Input | [107–113] | [+4] 3 systematic reviews, 4 retrospective studies | 0 | 0 | [-1] | 0 | [3] Moderate |
|  |  | 2 | There is an up-to-date list of network facilities in the same geographical area that provide referral care for women and children. | Input | [108,114] | [+4] 1 systematic review, 1 cross-sectional study | 0 | 0 | 0 | [-1] | [3] Moderate |
|  |  | 1 | The proportion of all newborns who died before or during transfer to a higher-level facility for further management. | Output | [109,115] | [+4] 1 systematic review, 1 retrospective study | 0 | 0 | 0 | 0 | [4] High |
|  |  | 2 | The proportion of all pregnant or postpartum women who died before or during transfer to a higherlevel facility for childbirth for further management. | Output | [108,109,116] | [+4] 3 systematic reviews | 0 | 0 | 0 | 0 | [4] High |
| 3.3 | For every woman and newborn referred within or between health facilities, there is appropriate information exchange and feedback to relevant health care staff. | 1 | The health facility has a standardized referral form to document relevant demographic and clinical information, which includes clinical findings, diagnosis, pre-referral interventions or treatment given and reason for referral. | Input | [117] | [+2] 1 cross-sectional study | 0 | 0 | 0 | [-1] | [1] Very low |
|  |  | 2 | The health facility has reliable communication methods, including a mobile phone, land line or radio, which is functioning at all times, for referrals and consultation on complicated cases. | Input | [108,118,119] | [+4] 2 systematic reviews, 1 RCT | 0 | 0 | [-1] | 0 | [3] Moderate |
|  |  | 3 | Evidence that the health facility has formal agreements, communication arrangements and a feedback system with referral centre(s). | Input | [120] | [+2] 1 cross-sectional study | 0 | 0 | 0 | [-1] | [1] Very low |
| 4.1 | All women and their families receive information about the care and have effective interactions with staff. | 1 | Easily understood health education materials, in an accessible written or pictorial format, are available in the languages of the communities served by the health facility. | Input | [121] | [+4] 1 systematic review | 0 | 0 | 0 | 0 | [4] High |
|  |  | 2 | Health care staff in the maternity unit are oriented and receive in-service training at least once every 12 months to improve their interpersonal communication and counselling skills and cultural competence. | Input | [122–124] | [+4] 3 systematic reviews | 0 | 0 | 0 | 0 | [4] High |
|  |  | 1 | The proportion of all women discharged from the labour and childbirth area of the facility who received written and verbal information and counselling on the following elements before discharge: nutrition and hygiene, birth spacing and family planning, exclusive breastfeeding and maintaining lactation, keeping their baby warm and clean, communication and play with the baby, danger signs for the mother and newborn and where to go in case of complications. | Output | [125,126] | [+4] 1 systematic review, 1 RCT | [-1] | 0 | 0 | [-1] | [2] Low |
|  |  | 2 | The proportion of all women who gave birth in the health facility who reported that they were given the opportunity to discuss their concerns and preferences. | Output | [127,128] | [+4] 2 systematic reviews | 0 | 0 | 0 | 0 | [4] High |
| 4.2 | All women and their families experience coordinated care, with clear, accurate information exchange between relevant health and social care professionals. | 1 | The health facility has a standard form for clinical progress notes and monitoring events during labour (partograph), birth and after birth to facilitate written hand-over. | Input | [129,130] | [+4] 2 systematic reviews | 0 | 0 | [-1] | [-1] | [2] Low |
|  |  | 2 | The health facility has written, up-to-date protocols for verbal and written hand-over of women and newborns at shift changes, during intra-facility transfer, on referral to other facilities and at discharge. | Input |  |  |  |  |  |  | No evidence found |
|  |  | 1 | The proportion of women attended during labour and childbirth for whom a partograph has been completed. | Output | [129,130] | [+4] 2 systematic reviews | 0 | 0 | [-1] | [-1] | [2] Low |
| 5.1 | All women and newborns have privacy around the time of labour and childbirth, and their confidentiality is respected. | 1 | The physical environment of the health facility allows privacy and the provision of respectful, confidential care, including the availability of curtains, screens, partitions and sufficient bed capacity. | Input | [127,131] | [+4] 1 systematic review; 1 cross sectional | [-1] | 0 | 0 | 0 | [3] Moderate |
|  |  | 2 | The health facility has written, up-to-date protocols to ensure privacy and confidentiality for all women and newborns in all aspects of care. | Input | [127,131] | [+4] 1 systematic review; 1 cross sectional | [-1] | 0 | 0 | 0 | [3] Moderate |
|  |  | 1 | The proportion of procedures in the health facility that require written consent for which there is an associated record of the woman's consent. | Output | [132–134] | [+4] 1 systematic review, 1 cross-sectional study, 1 qualitative study | 0 | [-1] | [-1] | [-1] | [1] Very low |
| 5.2 | No woman or newborn is subjected to mistreatment, such as physical, sexual or verbal abuse, discrimination, neglect, detainment, extortion or denial of services. | 1 | The health facility has written, up-to-date, zero-tolerance non-discriminatory policies with regard to mistreatment of women and newborns. | Input | [135] | [+4] 1 systematic review | [-1] | 0 | 0 | 0 | [3] Moderate |
|  |  | 2 | The health facility has a system whereby the mothers of small, sick newborns can be close to and nurse their babies. | Input | [22,136] | [+4] 1 systematic review, 1 retrospective study | 0 | 0 | 0 | [-1] | [3] Moderate |
|  |  | 3 | The fee structures for maternity and newborn care are equitable, affordable and clearly displayed. | Input | [135] | 4 1 systematic review | [-1] | 0 | 0 | 0 | [3] Moderate |
| 5.3 | All women can make informed choices about the services they receive, and the reasons for interventions or outcomes are clearly explained. | 1 | The health facility has a written, up-to-date policy for obtaining informed consent from women before examinations and procedures. | Input |  |  |  |  |  |  | No evidence found |
|  |  | 2 | The health facility has a standard informed consent form that helps health care staff to provide easily understandable information to women in order to obtain their fully informed consent. | Input | [132–134] | [+4] 1 systematic review, 1 cross-sectional study, 1 qualitative study | 0 | 0 | [-1] | [-1] | [2] Low |
|  |  | 3 | Health care staff in the health facility receive in-service training and supportive supervision in effective informed consent procedures and in women’s right to choose care at childbirth. Orientation is provided for new staff. | Input | [137–139] | [+4] 1 systematic review, 2 cohort studies | 0 | [-1] | [-1] | [-1] | [1] Very low |
|  |  | 1 | The proportion of procedures in the health facility that require written consent for which there is an associated record of consent signed by the woman or a family member. | Output | [132–134] | [+4] 1 systematic review, 1 cross-sectional study, 1 qualitative study | 0 | [-1] | [-1] | [-1] | [1] Very low |
| 6.1 | Every woman is offered the option to experience labour and childbirth with the companion of her choice. | 1 | The labour and childbirth areas are organized in such a way as to allow a physical private space for the woman and her companion at the time of birth. | Input | [127,131] | [+4] 1 systematic review; 1 cross sectional | [-1] | 0 | 0 | 0 | [3] Moderate |
|  |  | 2 | The health facility has a written, up-to-date protocol, which is explained to women and their families, to encourage all women to have at least one person of their choice, as culturally appropriate, with them during labour, childbirth and the immediate postnatal period. | Input |  |  |  |  |  |  | No evidence found |
|  |  | 1 | The proportion of all women who gave birth in the health facility who had a companion of their choice during labour and childbirth. | Output | [135] | [+4] 1 systematic review | 0 | 0 | [-1] | 0 | [3] Moderate |
| 6.2 | Every woman receives support to strengthen her capability during childbirth. | 1 | Health care staff in the labour and childbirth areas of the maternity unit were oriented in nonpharmacological and pharmacological pain relief and received in-service training or sessions at least once in the preceding 12 months. | Input |  |  |  |  |  |  | No evidence found |
| 7.1 | Every woman and child has access at all times to at least one skilled birth attendant and support staff for routine care and management of complications. | 1 | The health facility has skilled birth attendants available at all times, in sufficient numbers to meet the anticipated work load. | Input | [140,141] | [+4] 2 systematic reviews | 0 | 0 | 0 | 0 | [4] High |
|  |  | 2 | The health facility has a written, up-to-date staffing policy, listing the numbers, types and competence of staff, that is reviewed continuously according to the work load. | Input |  |  |  |  |  |  | No evidence found |
| 7.2 | The skilled birth attendants and support staff have appropriate competence and skills to meet requirements during labour, childbirth and the early postnatal period. | 1 | The health facility has a programme for continuing professional development and skills development for all skilled birth attendants and other support staff and conducts regular training. | Input | [121,142–144] | [+4] 4 systematic reviews | [-1] | 0 | 0 | 0 | [3] Moderate |
|  |  | 2 | The health facility has standard procedures and plans for recruitment, deployment, motivation (recognition and reward scheme) and retention of all staff. | Input |  |  |  |  |  |  | No evidence found |
|  |  | 1 | The proportion of skilled birth staff at the health facility who received a written job description on deployment to the facility. | Output | [145,146] | [+2] 1 cross-sectional, 1 qualitative study | 0 | 0 | 0 | [-1] | [1] Very low |
| 7.3 | The managerial and clinical leadership of every health facility is collectively responsible for creating and implementing appropriate policies and fosters an environment that supports facility staff in continuous quality improvement. | 1 | The health facility has a written, up-to-date plan for improving the quality of care and a patient safety programme. | Input |  |  |  |  |  |  | No evidence found |
|  |  | 2 | The health facility has a written, up-to-date leadership structure, with defined roles and responsibilities and lines of accountability for reporting. | Input | [141] | [+4] 1 systematic review | 0 | 0 | 0 | [-1] | [3] Moderate |
|  |  | 3 | The health facility has a designated quality improvement team and responsible personnel. | Input | [147] | [+4] 1 systematic review | [-1] | 0 | 0 | 0 | [3] Moderate |
| 8.1 | Water, energy, sanitation, hand hygiene and waste disposal facilities are functioning, reliable, safe and sufficient for the needs of staff, women and their families. | 1 | The health facility has a functioning source of safe water located on the premises that is adequate to meet all demands for drinking, personal hygiene, medical interventions, cleaning, laundry and cooking for use by staff, women, newborns and their families. | Input | [78] | [+4] 1 systematic review | 0 | 0 | 0 | [-1] | [3] Moderate (duplicate of 1.8) |
|  |  | 2 | The health facility has leak-proof, covered, labelled waste bins and impermeable sharps containers available in every treatment area, to allow segregation of waste into four categories: sharps, nonsharps infectious waste, general non-infectious waste (e.g. food, packaging) and anatomical waste (e.g. placenta). | Input | [82,83] | [+2] 2 cross-sectional studies | 0 | 0 | 0 | 0 | [2] Low |
|  |  | 3 | The health facility has at least one functioning hand hygiene station per 10 beds, with soap and water or alcohol-based hand rubs, in all wards. | Input |  |  |  |  |  |  | No evidence found |
|  |  | 4 | The health facility has energy infrastructure (e.g. solar, generator, grid) that can meet all the electricity demands of the facility and associated infrastructure at all times, with a back-up power source. | Input | [148,149] | [+4] 1 systematic rev; 1 cross sectional study | 0 | 0 | 0 | [-1] | [3] Moderate |
| 8.2 | Areas for labour, childbirth and postnatal care are designed, organized and maintained so that every woman and newborn can be cared for according to their needs in private, to facilitate the continuity of care. | 1 | The health facility has a dedicated area in the labour and childbirth area for resuscitation of newborns, which is adequately equipped with a table or resuscitaire, radiant warmer, light and appropriate resuscitation equipment and supplies. | Input | [58,150] | [+4] 2 systematic reviews | 0 | 0 | 0 | 0 | [4] High |
|  |  | 2 | The health facility has a labour ward and an adequate number of birthing rooms or areas for the estimated number of births in the service area. | Input | [127,148,151] | [+4]  2 systematic reviews; 1 qualitative study | [-1] | 0 | 0 | [-1] | [2] Low |
|  |  | 3 | The health facility has clean, appropriately illuminated, well-ventilated labour, childbirth and neonatal areas and surroundings that allow for privacy and are adequately equipped, regularly cleaned and maintained. | Input | [127,131] | [+4] 1 systematic review; 1 cross sectional | [-1] | 0 | 0 | [-1] | [2] Low |
| 8.3 | Adequate stocks of medicines, supplies and equipment are available for routine care and management of complications. | 1 | The health facility has supplies of antihypertensive agents and magnesium sulfate in sufficient quantities, available at all times, in antenatal, labour, childbirth and postnatal areas for the management of women with pre-eclampsia. | Input | [27] | [+4] 1 systematic review | 0 | 0 | 0 | [-1] | [3] Moderate (duplicate of 1.2) |
|  |  | 2 | The health facility has uterotonic drugs and supplies for intravenous infusion (syringes, needles, infusion sets, intravenous fluid solutions and blood) available in sufficient quantities at all times in the childbirth and postnatal care areas for the management of women with postpartum haemorrhage. | Input | [32] | [+4]  1 systematic review | 0 | 0 | 0 | [-1] | [3] Moderate (duplicate of 1.3) |
|  |  | 3 | The health facility has supplies of antenatal corticosteroids (dexamethasone or betamethasone), antibiotics and magnesium sulfate available in sufficient quantities at all times to manage preterm births. | Input | [59–61] | [+4] (2 systematic reviews; 1 cohort study) | 0 | 0 | 0 | [-1] | [3] Moderate (duplicate of 1.6a) |

**Sources identified in rapid review**

[1] Jahn A, Iang MD, Shah U, et al. Maternity care in rural Nepal: a health service analysis. Tropical Medicine and International Health. 2000;5:657–665.

[2] Wako G, Berhane Y. Structural quality of reproductive health services in South-Central Ethiopia. Ethiopian Journal of Health Development. 2000;14:317–326.

[3] Mkoka DA, Goicolea I, Kiwara A, et al. Availability of drugs and medical supplies for emergency obstetric care: experience of health facility managers in a rural District of Tanzania. BMC Pregnancy and Childbirth. 2014;14:1–10.

[4] Mccormick ML, Sanghvi HCGU, Kinzie B, et al. Preventing postpartum hemorrhage in low-resource settings. International Journal of Gynecology & Obstetrics. 2002;77:267–275.

[5] Pantoja T, Abalos E, Chapman E, et al. Oxytocin for preventing postpartum haemorrhage ( PPH ) in non-facility birth settings ( Review ). Cochrane Database of Systematic Reviews. 2016;

[6] Blencowe H, Cousens S, Mullany LC, et al. Clean birth and postnatal care practices to reduce neonatal deaths from sepsis and tetanus: a systematic review and Delphi estimation of mortality effect. BMC Public Health. 2011;11:S11–S11.

[7] Himani BK, Kumar P. Effect of initiation of breast-feeding within one hour of the delivery on “maternal- infant bonding.” Nursing and Midwifery Research Journal. 2011;7:11.

[8] Debes AK, Kohli A, Walker N, et al. Time to initiation of breastfeeding and neonatal mortality and morbidity: a systematic review. BMC Public Health. 2013;13:S19–S19.

[9] NEOVITA Study Group. Timing of initiation, patterns of breastfeeding, and infant survival: prospective analysis of pooled data from three randomised trials. The Lancet Global Health. 2016;4:e266–e275.

[10] Christensson K, Siles C, Moreno L, et al. Temperature, metaboli adaptation and crying in healthy full-term newborns cared for skin-to-skin or in a cot. Acta Paediatr. 1992;81:488–493.

[11] Moore ER, Anderson GC. Randomized controlled trial of very early mother – infant skin-to-skin contact and breastfeeding status. Journal of Midwifery & Women’s Health. 2007;52:116–125.

[12] Moore E, Bergman N, Anderson G, et al. Early skin-to-skin contact for mothers and their healthy newborn infants (Review). Cochrane Database of Systematic Reviews. 2016;

[13] Hutton EK, Hassan ES. Late vs early clamping of the umbilical cord in full-term neonates: systematic review and meta-analysis of controlled trials. JAMA. 2007;297:1241–1252.

[14] Rabe H, Diaz-rossello J, Duley L, et al. Effect of timing of umbilical cord clamping and other strategies to influence placental transfusion at preterm birth on maternal and infant outcomes (Review). Cochrane Database of Systematic Reviews. 2012;

[15] Mcdonald S, Middleton P, Dowswell T, et al. Effect of timing of umbilical cord clamping of term infants on maternal and neonatal outcomes (Review). Evidence-Based Child Health. 2014;9:303–397.

[16] Dahm LS, James LS. Newborn temperature and calculated heat loss in the delivery room. Pediatrics. 1972;49:504–513.

[17] Hammarlund K, Nilsson G, Oberg P, et al. Transepidermal water loss in newborn infants. V. Evaporation from the skin and heat exchange during the first hours of life. Acta Paediatr Scand. 1980;69:385–392.

[18] Sobel HL, Silvestre MAA, Mantaring III JBV, et al. Immediate newborn care practices delay thermoregulation and breastfeeding initiation. Acta Paediatrica. 2011;100:1127–1133.

[19] Procianoy RS, Fernandes-Filho PH, Lazaro L, et al. The Influence of Rooming-in on Breastfeeding. Journal of Tropical Pediatrics. 1983;29:112–114.

[20] Norr KF, Roberts JE, Freese U. Early postpartum rooming-in and maternal attachment behaviors in a group of medically indigent primiparas. Journal of Nurse-Midwifery. 1989;34:85–91.

[21] Yamauchi Y, Yamanouchi I. The relationship between rooming-in/not rooming-in and breast-feeding variables. Acta Paediatr Scand. 1990;79:1017–1022.

[22] Jaafar S, Ho J, Lee K. Rooming-in for new mother and infant versus separate care for increasing the duration of breastfeeding (Review). Cochrane Database of Systematic Reviews. 2016;

[23] Puckett R, Offringa M. Prophylactic vitamin K for vitamin K deficiency bleeding in neonates (Review). Cochrane Database of Systematic Reviews. 2000;

[24] Ipema HJ. Use of oral vitamin K for prevention of late vitamin K deficiency bleeding in neonates when injectable vitamin K is not available. The Annals of Pharmacotherapy. 2012;46:879–883.

[25] Moss GD, Cartlidge PHT, Speidel BD, et al. Routine examination in the neonatal period. BMJ. 1991;302:878–879.

[26] Glazener CMA, Ramsay CR, Campbell MK, et al. Neonatal examination and screening trial (NEST): a randomised, controlled, switchback trial of alternative policies for low risk infants. BMJ. 1999;318:627–632.

[27] Duley L, Meher S, Jones L. Drugs for treatment of very high blood pressure during pregnancy (Review). Cochrane Database of Systematic Reviews. 2013;

[28] Duley L, Gülmezoglu A, Henderson-Smart D, et al. Magnesium sulphate and other anticonvulsants for women with pre-eclampsia (Review). Cochrane Database of Systematic Reviews. 2010;

[29] Duley L, Henderson-Smart D, Chou D. Magnesium sulphate versus phenytoin for eclampsia (Review). Cochrane Database of Systematic Reviews. 2010;

[30] Duley L, Henderson-Smart D, Walker G, et al. Magnesium sulphate versus diazepam for eclampsia (Review). Cochrane Database of Systematic Reviews. 2010;

[31] Abalos E, Duley L, Steyn D. Antihypertensive drug therapy for mild to moderate hypertension during pregnancy (Review). Cochrane Database of Systematic Reviews. 2014;

[32] Gulmezoglu AM, Souza JP, Mathai M. WHO Recommendations for the Prevention and Treatment of Postpartum Haemorrhage [Internet]. 2012. Available from: http://www.who.int/reproductivehealth/publications/maternal_perinatal_health.

[33] Bates I, Chapotera GK, Mckew S, et al. Maternal mortality in sub-Saharan Africa: the contribution of ineffective blood transfusion services. An International Journal of Obstetrics & Gynaecology. 2008;115:1331–1339.

[34] Ramani KV, Mavalankar DV, Govil D. Study of blood-transfusion services in Maharashtra and Gujarat States, India. Journal of Health, Population and Nutrition. 2009;27:259–270.

[35] Tunçalp Ö, Hofmeyr G, Gülmezoglu A. Prostaglandins for preventing postpartum haemorrhage (Review). Cochrane Database of Systematic Reviews. 2012;

[36] Hofmeyr G, Gülmezoglu A, Novikova N, et al. Postpartum misoprostol for preventing maternal mortality and morbidity (Review). Cochrane Database of Systematic Reviews. 2013;

[37] Mousa H, Blum J, Abou El Senoun G, et al. Treatment for primary postpartum haemorrhage (Review). Cochrane Database of Systematic Reviews. 2014;

[38] Campbell OMR, Graham WJ, The Lancet Maternal Survival Series steering group. Strategies for reducing maternal mortality: getting on with what works. Lancet. 2006;368:1284–1299.

[39] Yakoob MY, Ali MA, Ali MU, et al. The effect of providing skilled birth attendance and emergency obstetric care in preventing stillbirths. BMC Public Health. 2011;11:S7–S7.

[40] Duffy JMN, Mylan S, Showell M, et al. Pharmacologic intervention for retained placenta: a systematic review and meta-analysis. Obstetrics & Gynecology. 2015;125:711–718.

[41] Simonson C, Barlow P, Dehennin N, et al. Neonatal Complications of Vacuum-Assisted Delivery. Obstetrics & Gynecology. 2007;109:626–633.

[42] Johanson R, Menon V. Vacuum extraction versus forceps for assisted vaginal delivery (Review). Cochrane Database of Systematic Reviews. 2009;

[43] Nolens B, Lule J, Namiiro F, et al. Audit of a program to increase the use of vacuum extraction in Mulago Hospital, Uganda. BMC Pregnancy and Childbirth. 2016;16:1–8.

[44] Pereira C, Cumbi A, Malalane R, et al. Meeting the need for emergency obstetric care in Mozambique: work performance and histories of medical doctors and assistant medical officers trained for surgery. An International Journal of Obstetrics & Gynaecology. 2007;114:1530–1533.

[45] Pasha O, Mcclure EM, Wright LL, et al. A combined community- and facility-based approach to improve pregnancy outcomes in low-resource settings: a Global Network cluster randomized trial. BMC Medicine. 2013;11:1–12.

[46] Villar J, Carroli G, Zavaleta N, et al. Maternal and neonatal individual risks and benefits associated with caesarean delivery: multicentre prospective study. BMJ. 2007;335:1025.

[47] Alfirevic Z, Milan SJ, Livio S. Caesarean section versus vaginal delivery for preterm birth in singletons. Cochrane Database Syst Rev. 2012;6:CD000078.

[48] Pergialiotis V, Vlachos DG, Rodolakis A, et al. First versus second stage C/S maternal and neonatal morbidity: a systematic review and meta-analysis. European Journal of Obstetrics & Gynecology and Reproductive Biology. 2014;175:15–24.

[49] Boatin A, Cullinane F, Torloni M, et al. Audit and feedback using the Robson classification to reduce caesarean section rates: a systematic review. BJOG. 2017;1–7.

[50] Paxton A, Maine D, Freedman L, et al. The evidence for emergency obstetric care. International Journal of Gynecology & Obstetrics. 2005;88:181–193.

[51] Carmona F, Martínez-Román S, Manau D, et al. Immediate maternal and neonatal effects of low-forceps delivery according to the new criteria of The American college of Obstetricians and Gynecologists compared with spontaneous vaginal delivery in term pregnancies. American Journal of Obstetrics and Gynecology. 1995;173:55–59.

[52] O’Mahony F, Hofmeyr GJ, Menon V. Choice of instruments for assisted vaginal delivery. Cochrane Pregnancy and Childbirth Group, editor. Cochrane Database of Systematic Reviews [Internet]. 2010 [cited 2019 Jan 31]; Available from: http://doi.wiley.com/10.1002/14651858.CD005455.pub2.

[53] Nyamtema AS, Urassa DP, van Roosmalen J. Maternal health interventions in resource limited countries: a systematic review of packages, impacts and factors for change. BMC Pregnancy and Childbirth [Internet]. 2011 [cited 2019 Jan 31];11. Available from: https://bmcpregnancychildbirth.biomedcentral.com/articles/10.1186/1471-2393-11-30.

[54] Fraser W, Vendittelli F, Krauss I, et al. Effects of early augmentation of labour with amniotomy and oxytocin in nulliparous women: a meta-analysis. BJOG: An International Journal of Obstetrics & Gynaecology. 1998;105:189–194.

[55] Sadler LC, Davison T, McCowan LME. A randomised controlled trial and meta-analysis of active management of labour. BJOG: An International Journal of Obstetrics and Gynaecology. 2000;107:909–915.

[56] Dencker A, Berg M, Bergqvist L, et al. Early versus delayed oxytocin augmentation in nulliparous women with prolonged labour-a randomised controlled trial. BJOG: An International Journal of Obstetrics & Gynaecology. 2009;116:530–536.

[57] Donnell CPFO, Davis PG, Morley CJ. Positive pressure ventilation at neonatal resuscitation: review of equipment and international survey of practice. Acta Paediatr. 2004;93:583–588.

[58] Wall SN, Cc A, Niermeyer S, et al. Neonatal resuscitation in low-resource settings: What, who, and how to overcome challenges to scale up? International Journal of Gynecology and Obstetrics. 2009;107:S47-64.

[59] Crowley P. Prophylactic corticosteroids for preterm birth (Review). Cochrane Database of Systematic Reviews. 1996;

[60] Lee BH, Stoll BJ, Mcdonald SA, et al. Neurodevelopmental outcomes of extremely low birth weight infants exposed prenatally to dexamethasone versus betamethasone. Pediatrics. 2008;121:289–296.

[61] Mwansa-Kambafwile J, Cousens S, Hansen T, et al. Antenatal steroids in preterm labour for the prevention of neonatal deaths due to complications of preterm birth. International Journal of Epidemiology. 2017;Suppl 1:i122-133.

[62] Lawn JE, Mwansa-Kambafwile J, Horta BL, et al. ‘Kangaroo mother care’ to prevent neonatal deaths due to preterm birth complications. International Journal of Epidemiology. 2010;39:i144-154.

[63] Mccall E, Alderdice F, Halliday H, et al. Interventions to prevent hypothermia at birth in preterm and/or low birthweight infants (Review). Cochrane Database of Systematic Reviews. 2010;

[64] Conde-Agudelo A, Díaz-Rossello JL. Kangaroo mother care to reduce morbidity and mortality in low birthweight infants. Cochrane Neonatal Group, editor. Cochrane Database of Systematic Reviews [Internet]. 2016 [cited 2019 Jan 31]; Available from: http://doi.wiley.com/10.1002/14651858.CD002771.pub4.

[65] Swarnkar K, Vagha J. Effect of kangaroo mother care on growth and morbidity pattern in low birth weight infants. Journal of Krishna Institute of Medical Sciences University. 2016;5:91–99.

[66] Mercer BM, Arheart KL. Antimicrobial therapy in expectant management of preterm premature rupture of the membranes. Lancet. 1995;346:1271–1279.

[67] French L, Smaill FM. Antibiotic regimens for endometritis after delivery. In: The Cochrane Collaboration, editor. Cochrane Database of Systematic Reviews [Internet]. Chichester, UK: John Wiley & Sons, Ltd; 2004 [cited 2019 Jan 31]. Available from: http://doi.wiley.com/10.1002/14651858.CD001067.pub2.

[68] Costantine MM, Rahman M, Ghulmiyah L, et al. Timing of perioperative antibiotics for cesarean delivery: a metaanalysis. American Journal of Obstetrics and Gynecology. 2008;199:301.e1-301.e6.

[69] Tita ATN, Rouse DJ, Blackwell S, et al. Evolving Concepts in Antibiotic Prophylaxis for Cesarean Delivery: A Systematic Review. Obstet Gynecol. 2009;113:675–682.

[70] Kenyon S, Boulvain M, Neilson J. Antibiotics for preterm rupture of membranes (Review). Cochrane Database of Systematic Reviews. 2013;

[71] Buppasiri P, Lumbiganon P, Thinkhamrop J, et al. Antibiotic prophylaxis for third- and fourth-degree perineal tear during vaginal birth. Cochrane Pregnancy and Childbirth Group, editor. Cochrane Database of Systematic Reviews [Internet]. 2014 [cited 2019 Jan 31]; Available from: http://doi.wiley.com/10.1002/14651858.CD005125.pub4.

[72] Smaill F, Grivell R. Antibiotic prophylaxis versus no prophylaxis for preventing infection after cesarean section (Review). Cochrane Database of Systematic Reviews. 2014;

[73] Meaney-Delman D, Bartlett LA, Gravett MG, et al. Oral and Intramuscular Treatment Options for Early Postpartum Endometritis in Low-Resource Settings: A Systematic Review. Obstetrics & Gynecology. 2015;125:789–800.

[74] Thinkhamrop J, Hofmeyr G, Adetoro O, et al. Antibiotic prophylaxis during the second and third trimester to reduce adverse pregnancy outcomes and morbidity (Review). Cochrane Database of Systematic Reviews. 2015;

[75] Mtitimila E, Cooke R. Antibiotic regimens for suspected early neonatal sepsis (Review). Cochrane. 2009;

[76] Downie L, Armiento R, Subhi R, et al. Community-acquired neonatal and infant sepsis in developing countries: efficacy of WHO’s currently recommended antibiotics--systematic review and meta-analysis. Archives of Disease in Childhood. 2013;98:146–154.

[77] Ohlsson A, Shah VS. Intrapartum antibiotics for known maternal Group B streptococcal colonization. Cochrane Pregnancy and Childbirth Group, editor. Cochrane Database of Systematic Reviews [Internet]. 2014 [cited 2019 Jan 31]; Available from: http://doi.wiley.com/10.1002/14651858.CD007467.pub4.

[78] Chawla SS, Gupta S, Onchiri FM, et al. Water availability at hospitals in low- and middle-income countries: implications for improving access to safe surgical care. Journal of Surgical Research. 2016;205:169–178.

[79] Harhay MO, Halpern SD, Harhay JS, et al. Health care waste management: a neglected and growing public health problem worldwide. Tropical Medicine & International Health. 2009;14:1414–1417.

[80] Arshad N, Nayyar S, Amin DF, et al. Hospital Waste Disposal: A Review Article. J. Pharm. Sci. 2011;8.

[81] Hossain MdS, Santhanam A, Nik Norulaini NA, et al. Clinical solid waste management practices and its impact on human health and environment – A review. Waste Management. 2011;31:754–766.

[82] Patil GV, Pokhrel K. Biomedical solid waste management in an Indian hospital: a case study. Waste Management. 2005;25:592–599.

[83] Aziz AM, Ashton H, Pagett A, et al. Sharps management in hospital: an audit of equipment, practice and awareness. British Journal of Nursing. 2009;18:92–98.

[84] Dettenkofer M, Block C. Hospital disinfection: efficacy and safety issues: Current Opinion in Infectious Diseases. 2005;18:320–325.

[85] Allegranzi B, Gayet-Ageron A, Damani N, et al. Global implementation of WHO’s multimodal strategy for improvement of hand hygiene: A quasi-experimental study. The Lancet Infectious Diseases. 2013;13:843–851.

[86] Moghnieh R, Soboh R, Abdallah D, et al. Health care workers’ compliance to the My 5 Moments for Hand Hygiene: Comparison of 2 interventional methods. American Journal of Infection Control. 2017;45:89–91.

[87] Pereira EB, Jorge MT, Oliveira EJ, et al. Evaluation of the Multimodal Strategy for Improvement of Hand Hygiene as Proposed by the World Health Organization. Journal of Nursing Care Quality. 2017;32:E11–E19.

[88] Zaidi AK, Huskins WC, Thaver D, et al. Hospital-acquired neonatal infections in developing countries. The Lancet. 2005;365:1175–1188.

[89] Srivastava S, Shetty N. Healthcare-associated infections in neonatal units: lessons from contrasting worlds. Journal of Hospital Infection. 2007;65:292–306.

[90] Rosenberg KD, Eastham CA, Kasehagen LJ, et al. Marketing Infant Formula Through Hospitals: the Impact of Commercial Hospital Discharge Packs on Breastfeeding. Am J Public Health. 2008;98:290–295.

[91] Semenic S, Childerhose JE, Lauzière J, et al. Barriers, Facilitators, and Recommendations Related to Implementing the Baby-Friendly Initiative (BFI): An Integrative Review. Journal of Human Lactation. 2012;28:317–334.

[92] Piwoz EG, Huffman SL. The Impact of Marketing of Breast-Milk Substitutes on WHO-Recommended Breastfeeding Practices. Food and Nutrition Bulletin. 2015;36:373–386.

[93] Carroli G, Mignini L. Episiotomy for vaginal birth (Review). Cochrane Database of Systematic Reviews. 2012;

[94] Pasha O, Saleem S, Ali S, et al. Maternal and newborn outcomes in Pakistan compared to other low and middle income countries in the Global Network’s Maternal Newborn Health Registry: an active, community-based, pregnancy surveillance mechanism. Reprod Health. 2015;12:S15.

[95] Frøen JF, Myhre SL, Frost MJ, et al. eRegistries: Electronic registries for maternal and child health. BMC Pregnancy Childbirth [Internet]. 2016 [cited 2019 Jan 31];16. Available from: https://www.ncbi.nlm.nih.gov/pmc/articles/PMC4721069/.

[96] Mangin D, Stephen G, Bismah V, et al. Making patient values visible in healthcare: a systematic review of tools to assess patient treatment priorities and preferences in the context of multimorbidity. BMJ Open [Internet]. 2016 [cited 2019 Jan 31];6. Available from: https://www.ncbi.nlm.nih.gov/pmc/articles/PMC4908882/.

[97] Fair M, Cyr M, Allen AC, et al. An assessment of the validity of a computer system for probabilistic record linkage of birth and infant death records in Canada. Chronic Diseases in Canada. 2000;21:8–13.

[98] Mikkelsen L, Phillips DE, AbouZahr C, et al. A global assessment of civil registration and vital statistics systems: monitoring data quality and progress. The Lancet. 2015;386:1395–1406.

[99] Pattinson R, Say L, Makin J, et al. Critical incident audit and feedback to improve perinatal and maternal mortality and morbidity ( Review ). Cochrane Database of Systematic Reviews. 2005;1–15.

[100] Biswas A, Rahman F, Eriksson C, et al. Facility Death Review of Maternal and Neonatal Deaths in Bangladesh. PLoS One [Internet]. 2015 [cited 2019 Jan 31];10. Available from: https://www.ncbi.nlm.nih.gov/pmc/articles/PMC4634754/.

[101] Korejo R, Bhutta S, Noorani KJ, et al. An audit and trends of perinatal mortality at the Jinnah Postgraduate Medical Centre, Karachi. :4.

[102] Lori JR, Rominski S, Osher BF, et al. A CASE SERIES STUDY OF PERINATAL DEATHS AT ONE REFERRAL CENTER IN RURAL POST-CONFLICT LIBERIA. Matern Child Health J [Internet]. 2014 [cited 2019 Jan 31];18. Available from: https://www.ncbi.nlm.nih.gov/pmc/articles/PMC3703491/.

[103] Kumar PP, Kumar CD, Shaik F, et al. Transported Neonates by a Specialist Team— How STABLE are they. Indian Journal of Pediatrics. 2011;78:860–862.

[104] Narang M, Kaushik JS, Sharma AK, et al. Predictors of mortality among the neonates transported to referral centre in Delhi, India. Indian Journal of Public Health. 2013;57:100–104.

[105] Rathod D, Adhisivam B, Bhat BV. Transport of sick neonates to a tertiary care hospital, south India: condition at arrival and outcome. Tropical Doctor. 2015;45:96–99.

[106] Sibley L, Sipe TA, Koblinsky M. Does traditional birth attendant training improve referral of women with obstetric complications: a review of the evidence. Social Science & Medicine. 2004;59:1757–1768.

[107] Mucunguzi S, Wamani H, Lochoro P, et al. Effects of Improved Access to Transportation on Emergency obstetric Care Outcomes in Uganda. :8.

[108] Murray SF, Pearson SC. Maternity referral systems in developing countries: Current knowledge and future research needs. Social Science & Medicine. 2006;62:2205–2215.

[109] Hussein J, Kanguru L, Astin M, et al. The Effectiveness of Emergency Obstetric Referral Interventions in Developing Country Settings: A Systematic Review. PLoS Med [Internet]. 2012 [cited 2019 Jan 31];9. Available from: https://www.ncbi.nlm.nih.gov/pmc/articles/PMC3393680/.

[110] Tayler‐Smith K, Zachariah R, Manzi M, et al. An ambulance referral network improves access to emergency obstetric and neonatal care in a district of rural Burundi with high maternal mortality. Tropical Medicine & International Health. 2013;18:993–1001.

[111] Wilson A, Hillman S, Rosato M, et al. A systematic review and thematic synthesis of qualitative studies on maternal emergency transport in low- and middle-income countries. International Journal of Gynecology & Obstetrics. 2013;122:192–201.

[112] Raj SS, Manthri S, Sahoo PK. Emergency referral transport for maternal complication: lessons from the community based maternal death audits in Unnao district, Uttar Pradesh, India. Int J Health Policy Manag. 2015;4:99–106.

[113] Tsegaye A, Somigliana E, Alemayehu T, et al. Ambulance referral for emergency obstetric care in remote settings. International Journal of Gynecology & Obstetrics. 2016;133:316–319.

[114] Nahar S, Banu M, Nasreen HE. Women-focused development intervention reduces delays in accessing emergency obstetric care in urban slums in Bangladesh: a cross-sectional study. BMC Pregnancy Childbirth. 2011;11:11.

[115] Awoonor-Williams JK, Bailey PE, Yeji F, et al. Conducting an audit to improve the facilitation of emergency maternal and newborn referral in northern Ghana. Global Public Health. 2015;10:1118–1133.

[116] Singh S, Doyle P, Campbell OM, et al. Referrals between Public Sector Health Institutions for Women with Obstetric High Risk, Complications, or Emergencies in India – A Systematic Review. PLoS ONE. 2016;11:1–23.

[117] Bansal R, Tandon H, Prateek S. Developing a two- way referral system for optimising antenatal care delivery. Bankok, Thailand; 2003.

[118] Lund S, Hemed M, Nielsen BB, et al. Mobile phones as a health communication tool to improve skilled attendance at delivery in Zanzibar: a cluster-randomised controlled trial. BJOG: An International Journal of Obstetrics & Gynaecology. 2012;119:1256–1264.

[119] Oyeyemi SO, Wynn R. The use of cell phones and radio communication systems to reduce delays in getting help for pregnant women in low- and middle-income countries: a scoping review. Glob Health Action [Internet]. 2015 [cited 2019 Jan 31];8. Available from: https://www.ncbi.nlm.nih.gov/pmc/articles/PMC4567587/.

[120] Nordberg E, Holmberg S, Kiugu S. Exploring the interface between first and second level of care: referrals in rural Africa. Tropical Medicine & International Health. 1996;1:107–111.

[121] Althabe F, Bergel E, Cafferata ML, et al. Strategies for improving the quality of health care in maternal and child health in low- and middle-income countries: an overview of systematic reviews. Paediatric and Perinatal Epidemiology. 2008;22:42–60.

[122] Dettrick Z, Firth S, Jimenez Soto E. Do Strategies to Improve Quality of Maternal and Child Health Care in Lower and Middle Income Countries Lead to Improved Outcomes? A Review of the Evidence. PLoS One [Internet]. 2013 [cited 2019 Jan 31];8. Available from: https://www.ncbi.nlm.nih.gov/pmc/articles/PMC3857295/.

[123] Lassi ZS, Middleton PF, Bhutta ZA, et al. Strategies for improving health care seeking for maternal and newborn illnesses in low- and middle-income countries: a systematic review and meta-analysis. Glob Health Action [Internet]. 2016 [cited 2019 Jan 31];9. Available from: https://www.ncbi.nlm.nih.gov/pmc/articles/PMC4864851/.

[124] Lassi ZS, Musavi NB, Maliqi B, et al. Systematic review on human resources for health interventions to improve maternal health outcomes: evidence from low- and middle-income countries. Hum Resour Health [Internet]. 2016 [cited 2019 Jan 31];14. Available from: https://www.ncbi.nlm.nih.gov/pmc/articles/PMC4789263/.

[125] Johnson A, Sandford J, Tyndall J. Written and verbal information versus verbal information only for patients being discharged from acute hospital settings to home (Review). Cochrane Database of Systematic Reviews. 2003;

[126] Kabakian-Khasholian T, Campbell OMR. Impact of written information on women’s use of postpartum services: a randomised controlled trial. Acta Obstetricia et Gynecologica. 2007;86:793–798.

[127] Srivastava A, Avan BI, Rajbangshi P, et al. Determinants of women’s satisfaction with maternal health care: a review of literature from developing countries. BMC Pregnancy and Childbirth. 2015;15:1–12.

[128] Bradley S, Mccourt C, Rayment J, et al. Disrespectful intrapartum care during facility-based delivery in sub-Saharan Africa: A qualitative systematic review and thematic synthesis of women’ s perceptions and experiences. Social Science & Medicine. 2016;169:157–170.

[129] Lavender T, Hart A, Smyth RM. Effect of partogram use on outcomes for women in spontaneous labour at term. Cochrane Pregnancy and Childbirth Group, editor. Cochrane Database of Systematic Reviews [Internet]. 2013 [cited 2018 Jun 18]; Available from: http://doi.wiley.com/10.1002/14651858.CD005461.pub4.

[130] Bedwell C, Levin K, Pett C, et al. A realist review of the partograph: when and how does it work for labour monitoring? BMC Pregnancy Childbirth [Internet]. 2017 [cited 2018 Jun 18];17. Available from: https://www.ncbi.nlm.nih.gov/pmc/articles/PMC5237234/.

[131] Lule GS, Tugumisirize J, Ndekha M. Quality of care and its effects on utilisation of maternity services at health centre level. East African Medical Journal. 2000;77:250–255.

[132] Williams MV, Parker RM, Baker DW, et al. Inadequate Functional Health Literacy Among Patients at Two Public Hospitals. JAMA. 1995;274:1677–1682.

[133] Schenker Y, Fernandez A, Sudore R, et al. Interventions to Improve Patient Comprehension in Informed Consent for Medical and Surgical Procedures: A Systematic Review. Medical Decision Making. 2011;31:151–173.

[134] Ebert L, Bellchambers H, Ferguson A, et al. Socially disadvantaged women’s views of barriers to feeling safe to engage in decision-making in maternity care. Women and Birth. 2014;27:132–137.

[135] Bohren MA, Vogel JP, Hunter EC, et al. The Mistreatment of Women during Childbirth in Health Facilities Globally: A Mixed-Methods Systematic Review. PLoS Med [Internet]. 2015 [cited 2018 Jun 18];12. Available from: https://www.ncbi.nlm.nih.gov/pmc/articles/PMC4488322/.

[136] Abecasis FDCG, Gomes A. Rooming-in for preterm infants: How far should we go? Five-year experience at a tertiary hospital. Acta Paediatrica. 2006;95:1567–1570.

[137] Dwamena F, Holmes-Rovner M, Gaulden CM, et al. Intervention for providers to promote a patient-centered approach in clinical consultations. Cochrane Database of Systematic Reviews. 2012;CD003267–CD003267.

[138] Abuya T, Warren CE, Miller N, et al. The effect of a multi-component intervention on disrespect and abuse during childbirth in Kenya. BMC Pregnancy and Childbirth. 2015;15:1–14.

[139] Kujawski SA, Freedman LP, Ramsey K, et al. Community and health system intervention to reduce disrespect and abuse during childbirth in Tanga Region, Tanzania : A comparative before-and-after study. PLoS Medicine. 2017;14:1–16.

[140] Gerein N, Green A, Pearson S. The Implications of Shortages of Health Professionals for Maternal Health in Sub-Saharan Africa. Reproductive Health Matters. 2006;14:40–50.

[141] Dogba M, Fournier P. Human resources and the quality of emergency obstetric care in developing countries: a systematic review of the literature. Human Resources for Health. 2009;17:1–12.

[142] Forsetlund L, Bjørndal A, Rashidian A, et al. Continuing education meetings and workshops: effects on professional practice and health care outcomes (Review). Cochrane Database of Systematic Reviews. 2009;

[143] Lonkhuijzen L van, Dijkman A, Roosmalen J van, et al. A systematic review of the effectiveness of training in emergency obstetric care in low-resource environments. BJOG: An International Journal of Obstetrics & Gynaecology. 2010;117:777–787.

[144] Opiyo N, English M. In-service training for health professionals to improve care of the seriously ill newborn or child in low and middle-income countries (Review). Cochrane Database of Systematic Reviews. 2010;

[145] Fort AL, Voltero L. Factors affecting the performance of maternal health care providers in Armenia. Human Resources for Health. 2004;2:1–11.

[146] Bradley S, McAuliffe E. Mid-level providers in emergency obstetric and newborn health care: factors affecting their performance and retention within the Malawian health system. Human Resources for Health. 2009;7:1–8.

[147] Raven J, Hofman J, Adegoke A, et al. Methodology and tools for quality improvement in maternal and newborn health care. International Journal of Gynecology and Obstetrics. 2011;114:4–9.

[148] Knight HE, Self A, Kennedy SH. Why Are Women Dying When They Reach Hospital on Time? A Systematic Review of the ‘Third Delay.’ PLoS ONE. 2013;8:1–9.

[149] Oguntunde O, Charyeva Z, Cannon M, et al. Factors influencing the use of magnesium sulphate in pre-eclampsia / eclampsia management in health facilities in Northern Nigeria: a mixed methods study. BMC Pregnancy and Childbirth. 2015;15:1–8.

[150] Lee ACC, Cousens S, Wall SN, et al. Neonatal resuscitation and immediate newborn assessment and stimulation for the prevention of neonatal deaths: a systematic review, meta- analysis and Delphi estimation of mortality effect. BMC Public Health. 2011;11:S12–S12.

[151] Matsuoka S, Aiga H, Chan L, et al. Perceived barriers to utilization of maternal health services in rural Cambodia. Health Policy. 2010;95:255–263.
