# Supplementary material for: Translating international guidelines for use in routine maternal and neonatal healthcare quality measurement
Source: Glob Health Action. 2020 Jul 13;13(1):1783956. doi: 10.1080/16549716.2020.1783956 (PMC7480423; doi:10.1080/16549716.2020.1783956)
Supplement: Supplemental Material [file ZGHA_A_1783956_SM0135.zip › MHQ Paper_SupplementaryFileTable3_2June2020.docx]

**Supplementary Data Table 3: List of all 56 indicators prioritized by the participants**

| **Priority level** | **Category** | **Type** | **QS*** | **#** | **Indicator** | **Participant comments** |
| --- | --- | --- | --- | --- | --- | --- |
| HIGH | Emergency Obstetric Care 1 | Input | 1.2 | 1 | The health facility has supplies of oral and intravenous antihypertensive agents and magnesium sulfate available in sufficient quantities at all times in the antenatal, labour and childbirth areas of the maternity unit. | Clear definition of "sufficient quantities" is needed |
| HIGH | Emergency Obstetric Care 1 | Input | 1.3 | 2 | The health facility has uterotonic drugs and supplies for intravenous fluid and blood administration (syringes, needles, intravenous cannulas, intravenous fluid solutions, blood) available in sufficient quantities at all times in the childbirth and postnatal care areas. | Clear definition of "sufficient quantities" is needed |
| HIGH | Emergency Obstetric Care 1 | Input | 1.3 | 3 | A functional blood transfusion service is available in the health facility at all times. | Applicable to facilities providing CEmOC |
| HIGH | Emergency Obstetric Care 1 | Output | 1.2 | 1 | The proportion of all women with severe pre-eclampsia or eclampsia in the health facility who received the full dose of magnesium sulfate. |  |
| HIGH | Emergency Obstetric Care 1 | Output | 1.2 | 2 | The proportion of all women with severe pregnancy-induced hypertension in the health facility who received the recommended antihypertensives. |  |
| HIGH | Emergency Obstetric Care 1 | Output | 1.3 | 1 | The proportion of all women with post-partum haemorrhage in the health facility who received therapeutic uterotonic drugs. |  |
| HIGH | Emergency Obstetric Care 1 | Output | 1.3 | 2 | The proportion of all women in the health facility with post-partum haemorrhage due to a retained placenta for whom manual removal of the placenta was performed by a skilled birth attendant. |  |
| HIGH | Emergency Obstetric Care 2 | Input | 1.4 | 2 | The health facility has the essential supplies and equipment for vacuum or forceps-assisted delivery, including newborn resuscitation equipment, available in sufficient quantities at all times in the childbirth area of the maternity unit. | Clear definition of "sufficient quantities" is needed |
| HIGH | Emergency Obstetric Care 2 | Input | 1.7a | 1 | The health facility has supplies of oral and injectable first- and second-line antibiotics (ampicillin or penicillin and gentamicin, clindamycin, cephalosporin and metronidazole) available in sufficient quantities at all times for the expected case load. | Clear definition of "sufficient quantities" is needed |
| HIGH | Emergency Obstetric Care 2 | Output | 1.1a | 2 | The proportion of all women who gave birth in the health facility who received oxytocin within 1 min of the birth of their baby | Would need observer to collect this time-sensitive data |
| HIGH | Emergency Obstetric Care 2 | Output | 1.4 | 2 | The proportion of all women in the health facility with prolonged and/or obstructed labour who gave birth by caesarean section. |  |
| HIGH | Emergency Obstetric Care 2 | Output | 1.4 | 3 | The proportion of all women who gave birth in the health facility who underwent instrumental vaginal birth for delayed second stage of labour. |  |
| HIGH | Emergency Obstetric Care 2 | Output | 1.4 | 5 | The proportion of all women in the health facility with confirmed delay in progress of the first stage of labour who received oxytocin for augmentation. |  |
| HIGH | Emergency Obstetric Care 2 | Output | 1.7a | 3 | The proportion of all women in the health facility with third- or fourth-degree perineal tears who received antibiotics. |  |
| HIGH | Emergency Obstetric Care 2 | Output | 1.9 | 1 | The proportion of all uncomplicated, spontaneous vaginal births in the health facility in which an episiotomy was performed. |  |
| HIGH | Emergency Newborn Care | Input | 1.1b | 2 | The health facility has supplies of sterile cord ties (or clamps) and scissors (or blades), available in sufficient quantities at all times for the expected number of births. | Clear definition of "sufficient quantities" is needed |
| HIGH | Emergency Newborn Care | Input | 1.5 | 1 | The health facility has a suction device, at least two sizes of neonatal mask and a self-inflating bag in the childbirth and neonatal areas of the maternity unit. |  |
| HIGH | Emergency Newborn Care | Input | 1.6a | 2 | The health facility has supplies of antenatal corticosteroids (dexamethasone or betamethasone), antibiotics and magnesium sulfate available in sufficient quantities at all times to manage preterm birth in accordance with WHO guidelines. | Clear definition of "sufficient quantities" is needed |
| HIGH | Emergency Newborn Care | Input | 1.6b | 2 | The health facility has supplies and materials to provide optimal thermal care to stable and unstable preterm babies, including kangaroo mother care (support binders, baby hats, socks), clean incubators and radiant warmers. |  |
| HIGH | Emergency Newborn Care | Input | 1.7b | 1 | The health facility has supplies of injectable antibiotics (at least first- and second-line antibiotics for neonatal sepsis and meningitis) available in sufficient quantities at all times for the expected case load. | Clear definition of "sufficient quantities" is needed |
| HIGH | Emergency Newborn Care | Input | 5.2 | 2 | The health facility has a system whereby the mothers of small, sick newborns can be close to and nurse their babies. |  |
| HIGH | Emergency Newborn Care | Input | 8.2 | 1 | The health facility has a dedicated area in the labour and childbirth area for resuscitation of newborns, which is adequately equipped with a table or resuscitaire, radiant warmer, light and appropriate resuscitation equipment and supplies. |  |
| HIGH | Immediate Newborn Care | Output | 1.8 | 2 | The proportion of newborns with suspected severe bacterial infection who received appropriate antibiotic therapy. |  |
| HIGH | Immediate Newborn Care | Output | 1.1b | 4 | The proportion of all newborns whose umbilical cord was clamped 1–3 min after birth. | Would need observer to collect this time-sensitive data |
| HIGH | Immediate Newborn Care | Output | 1.1b | 5 | The proportion of all newborns who were dried immediately and thoroughly at birth. |  |
| HIGH | Immediate Newborn Care | Output | 1.1b | 1 | The proportion of all newborns who were breastfed within 1 h of birth. | Would need observer to collect this time-sensitive data |
| HIGH | Immediate Newborn Care | Output | 1.1b | 2 | The proportion of all newborns who were kept in skin-to-skin contact (with body and head covered) with their mothers for at least 1 h after birth. | Would need observer to collect this time-sensitive data |
| HIGH | Immediate Newborn Care | Output | 1.1c | 1 | The proportion of all newborns on postnatal care wards or areas in the health facility who received vitamin K and full vaccination as per national guidelines. |  |
| HIGH | Immediate Newborn Care | Output | 1.1c | 4 | The proportion of all newborns in the health facility who received a full clinical examination before discharge. |  |
| HIGH | Infection Prevention/Facility Infrastructure | Input | 1.8 | 2 | The health facility ensures safe handling, storage and final disposal of infectious waste. |  |
| HIGH | Infection Prevention/Facility Infrastructure | Input | 1.8 | 1 | The health facility has a reliable water source on site and soap and towels (preferably disposable) or alcohol-based hand rub for hand hygiene. |  |
| HIGH | Infection Prevention/Facility Infrastructure | Input | 8.1 | 4 | The health facility has energy infrastructure (e.g. solar, generator, grid) that can meet all the electricity demands of the facility and associated infrastructure at all times, with a back-up power source. |  |
| HIGH | Referrals | Input | 3.2 | 1 | The health facility has ready access to a functioning ambulance or other vehicle for emergency transport of women and newborns to referral facilities. |  |
| HIGH | Referrals | Input | 3.3 | 2 | The health facility has reliable communication methods, including a mobile phone, land line or radio, which is functioning at all times, for referrals and consultation on complicated cases. |  |
| HIGH | Referrals | Output | 3.2 | 1 | The proportion of all newborns who died before or during transfer to a higher-level facility for further management. | Edit indicator to remove "during transfer"- not measurable |
| HIGH | Referrals | Output | 3.2 | 2 | The proportion of all pregnant or postpartum women who died before or during transfer to a higher level facility for childbirth for further management. | Edit indicator to remove "during transfer"- not measurable |
| HIGH | Data Systems | Input | 2.1 | 1 | The health facility has registers, data collection forms, clinical and observation charts in place at all time for routine recording and monitoring of all care processes for women and newborns. |  |
| HIGH | Data Systems | Input | 2.2 | 1 | The health facility has conducted reviews of maternal and perinatal deaths and near-misses at least once a month within the past six months and has a mechanism for implementing the recommendations of reviews. |  |
| HIGH | Staff & Training | Input | 4.1 | 2 | Health care staff in the maternity unit are oriented and receive in-service training at least once every 12 months to improve their interpersonal communication and counselling skills and cultural competence. |  |
| HIGH | Staff & Training | Input | 7.1 | 1 | The health facility has skilled birth attendants available at all times, in sufficient numbers to meet the anticipated work load. | Clear definition of "sufficient quantities" is needed |
| HIGH | Staff & Training | Input | 7.2 | 1 | The health facility has a programme for continuing professional development and skills development for all skilled birth attendants and other support staff and conducts regular training. | Specify exact type of trainings |
| HIGH | Staff & Training | Input | 7.3 | 2 | The health facility has a written, up-to-date leadership structure, with defined roles and responsibilities and lines of accountability for reporting. |  |
| HIGH | Staff & Training | Input | 7.3 | 3 | The health facility has a designated quality improvement team and responsible personnel. |  |
| MEDIUM | Data Systems | Output | 2.2 | 1 | The proportion of all perinatal deaths occurring in the health facility that were reviewed with standard audit tools. | Not currently practiced but important for quality monitoring |
| MEDIUM | Data Systems | Output | 2.2 | 2 | The proportion of all maternal deaths and near-misses occurring in the health facility that were reviewed with standard audit tools. | Not currently practiced but important for quality monitoring |
| MEDIUM | Respectful Care & Privacy | Input | 5.1 | 1 | The physical environment of the health facility allows privacy and the provision of respectful, confidential care, including the availability of curtains, screens, partitions and sufficient bed capacity. | Combine with QS 6.1 #1 |
| MEDIUM | Respectful Care & Privacy | Input | 5.2 | 1 | The health facility has written, up-to-date, zero-tolerance non-discriminatory policies with regard to mistreatment of women and newborns. | Combine with QS 5.1 #2 |
| MEDIUM | Respectful Care & Privacy | Input | 5.2 | 3 | The fee structures for maternity and newborn care are equitable, affordable and clearly displayed. |  |
| MEDIUM | Respectful Care & Privacy | Input | 6.1 | 1 | The labour and childbirth areas are organized in such a way as to allow a physical private space for the woman and her companion at the time of birth. | Combine with QS 5.1 #1 |
| MEDIUM | Respectful Care & Privacy | Output | 4.1 | 2 | The proportion of all women who gave birth in the health facility who reported that they were given the opportunity to discuss their concerns and preferences. |  |
| MEDIUM | Respectful Care & Privacy | Output | 6.1 | 1 | The proportion of all women who gave birth in the health facility who had a companion of their choice during labour and childbirth. |  |
| LOW | Emergency Obstetric Care 2 | Input | 1.7a | 2 | The health facility has written, up-to-date clinical protocols for treatment of women with, or at risk for, infections during labour, childbirth and the early postnatal period in the childbirth and postnatal care areas of the maternity unit that are consistent with WHO guidelines. | Adherence more important than presence of protocols |
| LOW | Emergency Obstetric Care 2 | Output | 1.9 | 2 | The proportion of women undergoing caesarean section in the health facility according to Robson classification groups. | Robson classification is not currently used in clinical practice in Bangladesh |
| LOW | Referrals | Input | 3.2 | 2 | There is an up-to-date list of network facilities in the same geographical area that provide referral care for women and children. | Physical list is not necessary as long as provider are aware of referral facilities |
| LOW | Respectful Care & Privacy | Input | 4.1 | 1 | Easily understood health education materials, in an accessible written or pictorial format, are available in the languages of the communities served by the health facility. | More important to retain QS 4.1 #2 |
| LOW | Respectful Care & Privacy | Input | 5.1 | 2 | The health facility has written, up-to-date protocols to ensure privacy and confidentiality for all women and newborns in all aspects of care. | Combine with QS 5.2 #1 |
