# Supplementary material for: Translating international guidelines for use in routine maternal and neonatal healthcare quality measurement
Source: Glob Health Action. 2020 Jul 13;13(1):1783956. doi: 10.1080/16549716.2020.1783956 (PMC7480423; doi:10.1080/16549716.2020.1783956)
Supplement: Supplemental Material [file ZGHA_A_1783956_SM0135.zip › MHQ Supplementary File Interview Guide_8May2020.docx]

***James P Grant School of Public Health, BRAC University, Bangladesh and***

***Metrics for Management, USA***

**Maternal & Neonatal Healthcare Quality Measurement: Real and Desired – Interview Guide**

**Introduction**

Thank you for agreeing to participate in this interview. You will help us assess how useful and feasible it is to measure maternal and newborn healthcare quality indicators in facilities like yours or those you support. There are no right or wrong answers. We are interested in hearing your opinion on this topic. This interview will be recorded using an audio device for research purposes, but all of your information will be kept private. If at any point during the interview you do not feel comfortable answering a question or wish to stop recording, please let us know.

**********************************************************************************

**General**

[Turn on the recorder.] First, I want to ask you some questions about your experience, and this facility.

1. Please tell me what your role is and how long you have been working both at this location and in this specific role.
2. [For non-providers/non-facility managers]: Please describe how you interact with facilities and providers to support monitoring of quality of maternal and newborn healthcare.
3. [Maternity ward]: Last month, how many deliveries took place in your maternity ward? Is this typical for the number of deliveries per month on average?

*Probe: Do all deliveries in the facility take place in the maternity ward? If not, where else do women deliver?*

- 1. Please take me through a typical experience of a mother delivering at your [facility/department].

*Probe: What are the admitting procedures? What are the routine checks at all stages of labor? How many doctors/nurses/midwives are present for a birth? How often does a clinician check a mother during the time she is in labor and immediately following delivery? How long after delivery is she typically kept at the facility?*

1. [Neonatal ward]: Last month, how many newborns were admitted to your ward? Is this typical for the number of newborn admissions per month on average?

*Probe: Are all newborns delivered at the facility admitted? Are there newborns delivered elsewhere who are admitted?*

- 1. Please take me through a typical experience of admitting a newborn to your [facility/department].

*Probe: What are the admitting procedures? What are the routine checks performed for a newborn? Who checks a newborn and how often? When is a newborn separated from the mother and for how long? How long is the newborn typically kept at the facility?*

1. Please describe how your [facility/department/organization] currently monitors [or supports the monitoring of] the quality of [maternal and/or newborn] healthcare provided.
   1. As part of your quality monitoring do you use a tool/form/checklist? Can you show it to me?
   2. What resources are needed for this/these quality checks?

*Probe: Personnel required to complete quality assessments and review results, time to complete, personnel training, frequency, cost*

*Are these personnel within or outside of the department? Are there ever situations when monitoring is done from someone outside of the facility?*

- 1. Can you describe what happens after any quality monitoring is done?

*Probe: Where is the information recorded? How, and with whom, is it shared?*

- 1. What are some of the challenges you see to ensuring high quality of care in these wards?

*Probe: What has been done to address those challenges?*

1. [For sister-in-charges] Please describe your role in managing and ordering supplies in your [facility/department].
   1. What are the challenges to making sure your [facility/department] has enough supplies?

*Probe: How often do you order? What informs your estimates/forecasts? How long does it take for supplies to arrive in your facility? Do you ever run out of supplies and if yes, how does this affect your work?*

**Specific Indicators**

[Present the list of indicators & list of questions to the respondent.] I am now going to show you lists of quality checks used in some facilities. I would like to understand if these checklist items are useful and feasible for [your facility/ facilities that you support]. Please refer to these lists of indicators and this list of questions for this part of our discussion.

1. Do you currently measure this indicator/ have this at your [facility or department or program]?

*Probe: Or do you measure part of this indicator?*

1. If yes, how is this information recorded, who monitors this, and how frequently?
2. How easy or difficult is it to collect this information, and why?
3. How useful is this information, and why?

*Probe: How is this information used?*

1. If no, what do you think about monitoring this indicator at your facility or department?
2. How easy or difficult would it be to collect this information, and why?
3. How useful do you think this information would be, and why?

*Probe: Would this information be used, and how?*

1. [For sister-in-charges] Would this indicator be useful when managing and ordering supplies?

**Other Indicators**

1. I see that in your checklist, you assess [other quality measures], which is not on our list. Can you tell me how this information is used and why it is important? Are there other quality measures that you currently monitor that are not on our lists of indicators?
   1. Please describe how this information is used and why this information is important.
2. If you did not have any constraints on resources (including resources like personnel, finances, physical space, or time), are there any quality measures you would like to measure that you currently do not?
   1. If yes, what quality measures would you want to monitor but currently do not?
      1. Please describe why this information is important and the challenges to collecting this information.

**Wrap Up**

1. Do you have any further information about [maternal and/or newborn] healthcare quality indicators in your [facility/department/program] that you would like to share?

This concludes the interview. Thank you for being available to speak with me today. [Turn off the recorder.]

**[Show these questions to the respondent during the discussion about specific indicators.]**

**Specific Indicator Questions**

1. Do you currently measure this indicator/ have this at your facility or department?

*Probe: Or do you measure part of this indicator?*

- 1. If **yes**, how is this information recorded, who monitors this, and how frequently?

1. How easy or difficult is it to collect this information, and why?
2. How useful is this information, and why?

*Probe: How is this information used?*

- 1. If **no**, what do you think about monitoring this indicator at your facility or department?

1. How easy or difficult would it be to collect this information, and why?
2. How useful do you think this information would be, and why?

*Probe: Would this information be used, and how?*
